# A Review of GC-Based Analysis of Non-Invasive Biomarkers of Colorectal Cancer and Related Pathways

**DOI:** 10.3390/jcm9103191

**Published:** 2020-10-01

**Authors:** Fernanda Monedeiro, Maciej Monedeiro-Milanowski, Tomasz Ligor, Bogusław Buszewski

**Affiliations:** 1 Department of Environmental Chemistry and Bioanalytics, Faculty of Chemistry, Nicolaus Copernicus University in Toruń, 87-100 Toruń, Poland; fmonedeiro@gmail.com (F.M.); msd2501@chem.uni.torun.pl (T.L.); 2 Interdisciplinary Centre of Modern Technologies, Nicolaus Copernicus University in Toruń, 87-100 Toruń, Poland; milanowski.maciej@gmail.com

**Keywords:** colorectal cancer, VOCs, biomarker, gas chromatography, profiling, breath, urine, feces, pathways

## Abstract

Colorectal cancer (CRC) is the third most commonly diagnosed cancer in the world. In Europe, it is the second most common cause of cancer-related deaths. With the advent of metabolomics approaches, studies regarding the investigation of metabolite profiles related to CRC have been conducted, aiming to serve as a tool for early diagnosis. In order to provide further information about the current status of this field of research, 21 studies were systematically reviewed, regarding their main findings and analytical aspects. A special focus was given to the employment of matrices obtained non-invasively and the use of gas chromatography as the analytical platform. The relationship between the reported volatile and non-volatile biomarkers and CRC-related metabolic alterations was also explored, demonstrating that many of these metabolites are connected with biochemical pathways proven to be involved in carcinogenesis. The most commonly reported CRC indicators were hydrocarbons, aldehydes, amino acids and short-chain fatty acids. These potential biomarkers can be associated with both human and bacterial pathways and the analysis based on such species has the potential to be applied in the clinical practice as a low-cost screening method.

## 1. Introduction

### 1.1. Colorectal Cancer Background

According to data regarding cancer burden in 2018 (GLOBOCAN 2018), colorectal cancer (CRC) is currently the third most incident cancer type in the word, with nearly 1.85 million cases and 881 thousand deaths worldwide. In Europe, it occupies the second place in the ranking of cancer occurrence and related deaths, with approximately half a million new cases registered and almost a quarter of a million associated deaths. Moreover, research on cancer progression predicts an increase of 75% in CRC cases over the next 20 years [1]. The global population over time has experienced significant changes in their habits, notably the prevalence of sedentarism, increased intake of dietary fat and processed food, and exposure to carcinogens, all risk factors in CRC [2]. Such context presents a complex perspective on CRC, also a from socioeconomic point of view, emphasizing the need for prevention strategies and promotion of early diagnosis.

It is observed that around 95% of colorectal neoplasms are adenocarcinomas and start as colonic adenomatous polyps [3]. Then, a series of genomic and molecular alterations induce the development of the malignancy in the colon [4]. CRC can be prevented if an intervention occurs leading to excision of the polyps and conduction of proper treatment; therefore, approaches directed towards an early detection of polyps and lesions, before these achieve the malignancy threshold, have substantial importance to reduce both CRC incidence and mortality [3].

### 1.2. Available Diagnostic Methods

The fecal occult blood test (FOBT), also known as the guaiac test, is generally applied for CRC screening. Nevertheless, this procedure presents relatively low sensitivity, which for this once-only test can be 50% or lower [5,6]. Additionally, FOBT is affected by the presence of interferers, is not specific for distal gut blood and may be insensitive to smaller bleedings. The antibody-based fecal immunochemical test (FIT) for hemoglobin is an improved alternative to FOBT, obtaining a sensitivity greater than 80% [6]. Notwithstanding, the verification of fecal blood can have a low impact on CRC primary assessment and is occasionally indicative of late stage cancer [7]. Currently, colonoscopy is described as the gold-standard screening procedure for CRC as it presents high sensitivity and specificity. However, colonoscopy is a costly and invasive procedure, limiting a patient’s access to the examination and resulting in poor compliance rates, aspects that hinder successful implementation of this test in CRC prevention [8,9]. Imaging exams have great reported efficiency, although also carry limitations regarding the cost of procedures and required exposure to radiation [10].

The group of currently available CRC biomarkers can be classified according to the affected biological matrices related to colorectal neoplasm. The most common are tumor, blood and stool biomarkers [11]. Moreover, molecular indicators can be grouped into three classes: prognostic, predictive and diagnostic markers [12]. Prognostic markers indicate the possible progression of the disease, such as: adenomatous polyposis coli (almost 100% of individuals develop CRC with this germ line mutation) [13,14], p53 (tumor suppressor p53 expression) [12], and epidermal growth factor receptor (EGFR; up to 80% over expression in CRC) [15]. Predictive indicators are used to foresee treatment measures to be taken on a patient. They include, e.g., Kirsten rat sarcoma viral oncogene (KRAS; more than 50% of CRC patients carry a mutant allele) [13,16], BRAF (a mutant KRAS gene, which encodes protein B-Raf, found in only 30–40% of the 90% of patients not affected by anti-EGFR therapy) [14,16], and COX-2 (Cyclooxygenase-2; the expression exhibited in 70% of CRC tumors) [12]. Risk stratification and early detection of polyps are provided by diagnostic markers, such as: insulin like growth factor binding protein 2 (IGFBP2; elevated levels in plasma and serum of CRC patients) [12,14], telomerase (an enzyme responsible for synthesizing DNA from chromosome ends for which an increase in activity was noticed for 90% of colorectal tumors) [17], and pyruvate kinase M2 (PKM2; a glycolytic pyruvate kinase isoenzyme increased in the stool of CRC subjects) [16]. Epi proColon^®^ (Epigenomics Inc., San Diego, CA, USA) is a commercially available test relying on the verification of methylated Septin-9 in DNA extracted from blood, by means of polymerase chain reaction (PCR) [18]. This genetic alteration is associated with the presence of CRC tissue. Studies showed that Epi proColon^®^ exam presented sensitivity and specificity ranging from 75 to 81% and from 96 to 99%, respectively [19]. Nevertheless, subsequent clinical trials demonstrated that test sensitivity was insufficient in case of asymptomatic cases and stage I CRC. Cologuard^®^ (Exact Sciences Corporation, Madison, WI, USA) is a stool-based presumptive test for CRC, based on the qualitative detection of fecal DNA markers. This exam presented to be superior to the FIT test, although its rate of detection was around 42% in cases of advanced adenomas [8]. Apart from the displayed limitations, these screening strategies tend to achieve wider acceptance among the population and can indicate the need for further colonoscopic investigation, aiding a more approachable monitoring of CRC.

### 1.3. Metabolomics Studies on CRC

Metabolomics science emerged as a new approach to study biological systems [20]. In a metabolomics workflow, biological samples are processed and comprehensively analyzed in terms of total metabolites, which can belong to a specific chemical class depending on the envisioned approach and the methodologies selected for sample preparation and pre-concentration. Measurements can involve different analytical platforms, with emphasis given to chromatographic techniques—able to resolve complex mixtures—coupled to mass spectrometry [21], such as gas chromatography-mass spectrometry (GC-MS) and liquid chromatography-mass spectrometry (LC-MS) [22,23,24].

Among the small metabolites, volatile organic compounds (VOCs) are metabolic products that can elicit diversified patterns that may represent very specific biochemical ongoing processes in the organism. Volatiles’ profiles have been studied in the context of several diseases, especially in exhaled breath, using GC-based analyses [25,26,27,28]. In this context, GC analysis is extremely relevant, because it encompasses the group of VOC metabolites, which cannot be properly assessed by LC.

Research on global molecular metabolites as potential markers of diseases is a very interesting approach for the design of methods directed towards the early diagnosis and evaluation of patient’s response to therapeutic intervention [20,29]. Molecular profiling presents promising perspectives towards clinical applications. The assessment of a set of metabolites has the possibility to provide information regarding simultaneous metabolic alterations, potentially offering a more accurate and detailed diagnosis, thus, it represents a great advance in personalized medicine [30].

Although contemporary, metabolomics-based methods still face several challenges, such as: the existence of a large body of variables that may impact the metabolic profile; the lack of standardization in workflow protocol and irreproducibility between studies that lead to varied panels of potential biomarkers. Therefore, a deeper inspection is required in order to compare the results reported so far by different research groups concerning the metabolomic investigation in CRC, listing the main developments made to date, and thus offering insights into new aspects to be studied regarding CRC characterization.

This review aims to compile data obtained from different GC-based investigations on molecular metabolites of CRC in biological samples obtained non-invasively (breath, urine and feces). Considering this, the analytical aspects involved in the profiling studies and their main findings are described. Furthermore, candidate CRC biomarkers and their related origins are discussed from the point of view of cancer physiopathology. In this sense, the present review is directed to researchers working in the medical and metabolomics fields aiming to have an overview of explored analytical protocols and to consult the main contribution of such metabolic patterns on the elucidation of CRC-related mechanisms and their potential regarding disease diagnosis.

## 2. Studies on Colorectal Cancer Metabolic Biomarkers

### 2.1. Applied Methodologies

A critical matter involving metabolomics studies is the employment of varied protocols covering sample collection, processing and analysis. In this sense, the selection of specific analytical parameters can deeply influence the set of acquired metabolites, turning valid the discussion on the main aspects prevalent in sample pre-treatment, extraction procedure and analysis in GC-based metabolomics directed towards CRC markers investigation. Several techniques have been employed for the extraction and pre-concentration of the metabolites of interest in different biological samples. The particular characteristics of each matrix determine which sample preparation techniques are required, which in turn, have associated advantages and limitations to be observed by the analyst. Fundamental aspects regarding the selection of biological matrix are the concentration range of the target analytes in the sample, window of detection provided, matrix complexity and involved distribution mechanisms. Sample preparation techniques to be used should be chosen based on their ability to pre-concentrate the analyte, the availability of specific materials, required processing time and involved costs. Data concerning sample preparations details, study design and statistical approaches employed by the reviewed studies are summarized in Table 1.

### 2.2. General Analytical Platforms Available for Metabolomics Studies

There are many analytical platforms for molecular biomarker analysis, such as nuclear magnetic resonance (NMR), high performance liquid chromatography-mass spectrometry (HPLC-MS), ultrahigh performance liquid chromatography-mass spectrometry (UHPLC-MS), supercritical fluid chromatography-mass spectrometry (SFC-MS), capillary electrophoresis-mass spectrometry (CE-MS) and GC-MS [52,53]. NMR is highly reproducible technique which requires low amounts of the sample, enables quantitation without standards and allows for the identification of both polar and nonpolar compounds. The main disadvantage of NMR compared with MS is poor sensitivity, limiting analyses to low-abundant metabolites [52]. UHPLC-MS is more sensitive than HPLC-MS and requires smaller sample volume for injection. Recent UHPLC-MS are using porous particles with internal diameters smaller than 2 µm, which provide higher peak capacity, increased specificity and high-throughput capabilities as compared to HPLC columns [53]. The difficulty is that electrospray ionization (ESI) mass spectral libraries are not standardized like in the case of GC-MS [54]. SFC-MS is a promising tool in the field of metabolomics, next to GC and LC. It can analyze both polar and nonpolar compounds using “green” and rather cheap CO_2_ as the mobile phase [52]. CE-MS has low sensitivity and enables the analysis of polar compounds. CE-MS offers high-analyte resolution and a small volume of sample for injection (1–20 nL) [52,53]. GC-MS is a gold standard for the analysis of volatiles and it is, relatively, a simpler technique than LC-MS, more cost effective and with reduced matrix effect enabling to quantify compounds in picograms and identification using easy-accessible reference libraries [27,28,29]. It is also possible to separate and analyze semi-volatiles, for example when using solvent extraction as the sample preparation technique. However, most compounds require a chemical derivatization step at room or elevated temperatures to provide necessary volatility and thermal stability [54]. GC-MS is one of most important methods to analyze VOCs from various matrices like breath, saliva, urine, feces and blood for metabolomic purposes [55].

### 2.3. Search Strategy

On 20 May 2020, a literature search was performed in the electronic database Web of Science Core Collection (from Clarivate Analytics; Philadelphia, PA, USA), as well as Google Scholar. The used search terms were: colorectal cancer, volatile organic compounds, gas chromatography, breath, urine and feces, with time span from 2010 to 2020. Due to the historical relevance, two older articles involving breath samples (from 1977 and 1984) were also included.

Studies that were included met the following criteria; (i) at least two patient groups; one group with colon or digestive tract cancer in any stage and another group without cancer; (ii) analytical platform based on GC coupled with MS or other commonly used detectors; (iii) detection and identification of chemicals or gases in breath, urine and feces.

The following information was gathered from the articles, per type of matrix: author, year of publication, analytical method, method of data analysis, sample preparation technique, type of used GC column, patient groups and number of volunteers, degree of validation of the method and its level of sensitivity, specificity, accuracy and other statistical parameters.

Twenty-one studies on CRC molecular markers were reviewed, all of them employing gas chromatography as the analytical technique and comprising the investigation of urine, breath or fecal samples as sources of metabolites. Data regarding candidate CRC biomarkers and studied biological matrices are presented in Table 2—these compounds are referred to as being the most relevant for being reported as possible markers by more than one study and/or for the possibility of being addressed to formerly described biochemical mechanisms. Table S1 (Supplementary Materials) is a full, extended version of Table 2.

### 2.4. CRC Biomarkers in Urine

Qiu et al., 2010 profiled urine samples from the same group of patients (60 CRC diseased individuals with different stage of cancer) from previous serum experiments and 63 healthy volunteers. Patients were also examined before and after surgical operation in order to evaluate changes in volatile profiles. The postoperative urine specimens were collected on the seventh day after surgery. Authors used GC-MS with solvent extraction and derivatization (using ethyl chloroformate) of samples. In a predictive model, 187 volatile metabolites were found in 90% of samples allowing discrimination of CRC patients from the healthy controls in the predictive component. 16 potential biomarkers of disease between preoperative CRC patients and healthy controls were identified including decreasing compounds, e.g., 3-methylhistidine, histidine, citric acid, and increasing volatiles such as 5-hydroxytryptophan, 5-oxoproline, *p*-cresol and phenyl acetate. Four compounds, 5-hydroxytryptophan, 2-hydroxyhippuric acid, succinic acid and phenylacetylglutamine, demonstrated recovering tendency to healthy controls in the postoperative samples. Different CRC stages could be distinguished by six metabolites with characteristic expression levels. Levels of indoleacetic acid were elevated for stage I patients, p-hydroxyphenylacetic acid for stage II and 5-hydroxyindoleacetic acid for stage III. The highest concentration of 2-methylpropanoic acid was found for stage II with a sharp decrease for stage IV patients. A continuous increase in glutamic acid levels was observed until stage IV. Finally, stage I was characterized by proportionally large amount of leucine. Twenty-one VOCs, mostly amino acids and phenyl-containing constituents, allowed for discrimination between preoperative and postoperative states of patients and they are likely related to the metabolic changes resulting from the surgical operation [31].

Volatile metabolites from urine of 54 subjects were investigated in a study by Silva et al., 2011. Thirty-three cancer patients (oncological group: 14 leukaemia, 12 colorectal and 7 lymphoma) and 21 healthy (cancer-free) volunteers were enrolled in the experiment. Positive rates of 16 volatiles among the 82 detected were found to be statistically different (*p* < 0.05). They used an optimized technique relying on dynamic solid-phase microextraction in headspace mode (dHS-SPME), in combination with GC-MS-based metabolomics. Prior optimization concerned extraction time and extraction temperature and selection of SPME fiber. Oncological groups were characterized with the predominance of benzene derivatives, terpenoids and phenols. Levels of p-cymene, anisole, γ-terpinene, bornylene, dimethyl disulfide, 4-methylphenol, 1,2-dihydro-1,1,6-trimethylnaphthalene, 1,4,5-trimethylnaphthalene and 2,7-dimethylquinoline were elevated in colorectal patients than in lymphoma and leukaemia patients. Decreasing concentrations for CRC patients were observed for e.g., 1-octanol, heptanal, hexanal and dimethyl disulfide in comparison to healthy controls; 4-methyl-2-heptanone was only identified in colorectal patients [32].

Solvent extraction and derivatization combined with GC- time-of flight mass spectrometry (TOFMS) was used by Cheng et al., 2012, to find metabolite markers of colorectal cancer. A cohort of 103 CRC patients and 101 healthy subjects participated in the study. From the total 163 volatiles detected, 19 metabolites were selected as potential biomarkers based on statistical analysis by uni- and multivariate statistical methods. Using a set of seven metabolites, citric acid, hippuric acid, *p*-cresol, 2-aminobutyric acid, myristic acid, putrescine, and kynurenate, it was possible to discriminate CRC subjects from healthy volunteers, presenting AUROC (area under the receiver operating characteristic curve) of 0.993, sensitivity of 97.5% and specificity of 97.6% for the training set, and an area under the curve (AUC ) of 0.998, sensitivity of 97.5% and specificity of 100% for the testing set [33].

A field asymmetric ion mobility spectrometer (FAIMS) was used by Arasaradnam et al., 2014, for analyses of urine samples from 83 CRC patients and 50 healthy controls. Data analysis for FAIMS results was performed using fisher discriminant analysis. The sensitivity and specificity of FAIMS was 88% and 60%, respectively, for CRC and this technique allowed for the differentiation between CRC patients and healthy ones. The author conducted a parallel in-tube extraction (ITEX)-GC-MS experiment with the same samples. No unique chemical was identified in those with CRC compared with healthy volunteers. According to the incorporated table, they found nine different volatiles associated with colorectal cancer and they could be assigned to 26 NIST library targets for these peaks. There was no information if concentrations of these compounds changed, nevertheless they were included to Table 2 [34].

Liesenfeld et al., 2015, investigated urine samples from a cohort of 170 subjects divided in four groups: pre-surgery (79), post-surgery within few days (9), after 6 months follow-up (46) and after 12 months follow-up (36) CRC patients. A total of 82 VOCs detected by GC-MS were significant and allowed to distinguish pre- from post-surgery CRC patients. However, only 49 metabolites were included in Table 2, since the remaining VOCs were unknown (level three or four identification). Liesenfeld et al., 2015, attributed the origin of many significant metabolites to alternations of the gut microbiome affected by CRC surgery, such as 2,3-butanediol, pyrogallol, hydroquinone and maleamic acid. A total of 10 compounds (four identified) were highlighted as metabolites significant to discriminate pre-surgery CRC patients by disease stage. Levels of a dipeptide of hydroxyproline (Hyp-Hyp) and *p*-cresol-β-O-glucuronide were considerably elevated for intermediate (stage II–III) stage patients. For early (stage zero to 1) stage both mentioned compounds and hippuric acid and *p*-cresol were moderately elevated, while for late-stage CRC patients, their concentrations were diminished. The authors constructed a multivariate model containing 20 marker metabolites to differentiate the metabolite profiles of CRC patients prior to surgery from those at any post-surgery timepoint (AUCROC curve = 0.89; 95% CI:0.84–0.95) [35].

Delphan et al., 2018, used the same techniques as Liesenfeld et al., 2015, to obtain urine profiles from 163 CRC pre-surgery patients, 83 with 6 months follow-up and 54 with 12 months follow-up. The GC-MS and ^1^H-NMR experiments identified six compounds of interest: isoleucine, leucine, valine, (2Z)-3-methylglutaconic acid, 2-ethylhydracrylic acid and 2-methyl-3-hydroxybutyric acid. Elevated concentrations of 2-ethylhydracrylic acid and 2-methyl-3-hydroxybutyric acid were significantly associated with overall survival after 24 months of follow-up, independent of stage of CRC [36].

The FAIMS technique was used once again for development of VOC-based screening tool for CRC and adenomas (Mozdiak et al., 2019). Moreover, gas chromatography coupled with ion mobility spectrometry (GC–IMS) was also employed. A total of 163 patients were enrolled in the study. For patients grouped into categories according to diagnosis, FAIMS analysis revealed high sensitivity and specificity between CRC vs. normal control: 1.0 (95% CI 0.74–1) and 0.92 (95% 0.62–1), respectively. For GC-IMS study, the corresponding values showed a high degree of separation with a sensitivity of 0.80 (95% CI 0.44–0.97) and specificity of 0.83 (95% CI 0.63–0.95). However, when considering CRC cases grouped with adenomas and compared with other groups, the accuracy dropped significantly. Hence, urinary VOC profiles from CRC patients in combination with other (non-neoplastic) gastrointestinal disorders, are not sufficiently distinct to allow correct classification. No unique VOC biomarkers were found using GC-IMS. Summarizing, VOC signatures enabled correct classification of malignant patients from pre-malignant ones with higher test uptake and superior sensitivity than FOBT used for bowel cancer screening [37].

### 2.5. CRC Biomarkers in Feces

In studies regarding fecal samples, profiling of gut microflora is recurrent, in order to establish correlations between fecal metabolites and human microbiota. Previous evidence demonstrates differences between the microbial composition found in samples of heathy and CRC patients, supporting the existence of oncogenic bacteria, which potentially promote CRC initiation and tumor development [56]. Hence, the detection of specific bacterial metabolites, especially in feces, presented to be relevant in the assessment of CRC risk.

Feces samples were investigated by Weir et al., 2013, from healthy adults (*n* = 10) and colorectal cancer patients (*n* = 11). Using solvent extraction combined with GC-MS, they examined stool samples to find overall metabolite profiles and to extract short chain fatty acids (SCFAs). Microbial diversity in stool biota from CRC subjects and controls were identified using amplification of the V4 region of the bacterial 16S rRNA gene and pyrosequencing. Fourteen bacterial species (mostly butyrate-producing) were significantly more abundant in the stool of healthy individuals compared to CRC patients. On the other hand, four bacterial species were significantly over-represented in stool samples from CRC patients. The last mucin-degrading bacteria, *Akkermansia muciniphila*, was observed in significantly greater proportion in CRC stool samples. SCFA analysis resulted in finding six bacterially produced fatty acids that differed significantly between stool of healthy adults and subjects with CRC. Levels of acetic acid, propionic acid, valeric acid, and particularly isobutyric acid and isovaleric acid were significantly elevated for CRC samples. Butyric acid concentrations were higher in samples from controls. Independently, Weir et al., 2013, detected 27 global stool metabolites which they proposed as cancer biomarkers. Eleven of them were amino acids demonstrating a 41–80% increase in fecal samples from CRC patients, originating possibly from degradation of dietary proteins and intestinal mucins, which is consistent with the presence of bacteria *A. muciniphila*. Higher levels of glycerol, several unsaturated fatty acids and ursodeoxycholic acid (UDCA) were observed for healthy adults. Pearson’s correlations between groups of metabolites and bacterial genera/species revealed strong correlations between *Bacteroides finegoldii*, two *Dialister spp.*, and *Pseudobutyrivibrio ruminis* and increased stool free fatty acids and glycerol, as well as between *Ruminococcus spp.* and UDCA. A strong correlation existed also between bacterial genera *Phascolarctobacterium* and *Acidiminobacter* (noteworthy for CRC samples) and amino acids phenylalanine and glutamic acid [38].

In the study of Phua et al., 2014, GC-TOFMS was employed for the metabolic profiling of fecal samples from 11 CRC patients and 10 healthy subjects and to find metabolite markers from tumor specimens against their matched normal mucosae from eight CRC patients and 10 controls. The discrimination between CRC patients and healthy controls was evident based on fecal profiles (orthogonal partial least squares discriminant analysis (OPLS-DA), one predictive and three Y-orthogonal components, R^2^X = 0.373, R^2^Y = 0.995, Q^2^ (cumulative) = 0.215). The robustness of the OPLS-DA model was demonstrated by an AUROC of one. GC-TOFMS profiling also enabled the separation of tumor tissue from matched normal mucosae and to assign nine potential biomarkers of CRC. Glucose, galactose, 3-phosphoglycerate, citric acid, inosine, and creatinine were lower in CRC while uracil, uridine, and proline were significantly higher in tumor tissue. Three fecal markers were found to be also lower compared to healthy stool samples, namely nicotinic acid, fructose and linoleic acid. Authors pointed out that the conducted study revealed the ability to differentiate CRC subjects from healthy subjects regardless of the presence of samples containing blood beyond 1 mg Hb/g stool [39].

Bond et al., 2016, used SPME-GC-MS to analyze volatiles in stool samples of 137 participants, consisting of 60 controls, 56 patients with adenomatous polyp/s and 21 CRC patients. Four compounds were assigned as biomarkers, however their names were not mentioned due to alleged potential future intellectual property. After a tenfold cross validation, an AUC of 0.82 with a sensitivity of 87.9% and a specificity of 84.6% was measured [40].

Wang et al., 2017, applied very similar approach to Weir et al., 2013. A total of 27 subjects (15 CRC patients and 12 controls) were enrolled in the study. Cancer group consisted of stage II in four cases, stage III in six cases and stage IV in five clinical cases. Two parallel experiments involving GC-MS were conducted: global metabolite profiling and SCFA analysis. Bacterial species present in stool samples were identified using pyrosequencing for specific detection of the V4 region of bacterial 16S ribosomal RNA on the isolated genomic DNA. Eighteen bacteria obtained from gut flora differed significantly between CRC and control group. VOC profiling revealed 24 volatiles being proposed as markers of disease. Among them, the levels of four SCFAs (acetic acid, valeric acid, butyric acid and isovaleric acid) were elevated for CRC patients, while the concentration of isobutyric acid was diminished for the cancer group. Compounds with decreased levels for CRC samples included fatty acids (oleic acid, elaidic acid, linoleic acid and myristic acid), ursodeoxycholic acid and pantothenic acid (vitamin B_5_). The largest group of increased species were amino acids, represented by i.e., glutamic acid, leucine, serine, valine and phenylalanine. High positive correlations were observed between bacterial species and volatiles, including: *Bacteroides*, *Dialister* and *Pseudobutyrivibrio*—free fatty acids; *Ruminococcus*—ursodeoxycholic acid; *Phascolarctobacterium* and *Acidiminobacter*—phenylalanine and glutamic acid [41].

Fecal fatty acids acid profiles of CRC patients and healthy controls were analyzed by Song et al., 2018. A total of 54 subjects (26 CRC patients and 28 controls) participated in the study. Fecal samples were dedicated for two independent experiments, consisting in profiling of long- and short-chain fatty acids by solvent extraction with GC-MS. The most predominant saturated fatty acids among both gender groups were palmitic acid (C16:0), stearic acid (C18:0), and myristic acid (C14:0). The significant changes between profiles were observed only for the male group, no difference was estimated between CRC patients and healthy controls in the female group. The levels of total monounsaturated fatty acid (MUFA) and total omega-6 polyunsaturated fatty acids (PUFAs) were higher in the male CRC group than healthy controls. The differences were especially significant for two compounds, namely oleic acid (C18:1ω-9) and linoleic acid (C18:2ω-6). The levels of four SCFAs, acetic acid, butyric acid, propionic acid and valeric acid, were not considerably distinct between the controls and positive cohort [42].

HS-SPME-GC-MS was used by Bond et al., 2019, to identify volatiles from feces of 21 CRC patients, 56 subjects with adenomatous polyp(s) and 60 healthy controls. A total of 162 compounds were identified in whole sample set with eight volatiles proposed as CRC biomarkers. Seven were positively associated with CRC, namely propan-2-ol, hexan-2-one, ethyl 3-methylbutanoate, propan-2-yl butanoate, propan-2-yl pentanoate, 1,4-xylene, and propan-2-yl propanoate. Only one compound was negatively correlated with CRC—5-methyl-2-propan-2-yl-cyclohexan-1-ol (other name: dl-menthol). Propan-2-ol was pointed out as the most valuable single biomarker of disease with an AUROC of 0.76, a sensitivity of 83%, and a specificity of 71%. Meanwhile, 3-methylbutanoic acid and propan-2-ol in combination gave the best results: AUROC was 0.86, sensitivity 87.9% (95% CI 0.87–0.99) and specificity 84.6% (95% CI 0.65–1.0). Finally, a panel of three VOCs (propan-2-ol, hexan-2-one and ethyl 3-methylbutanoate) was evaluated as a key predictor of cancer using logistic regression analysis with value of AUROC equal to 0.73. The presence of all three mentioned VOCs indicates a person with CRC with six times bigger probability of having the disease [43].

### 2.6. CRC Biomarkers in Exhaled Breath

The first studies concerning colorectal cancer biomarkers in exhaled breath involved the determination of methane levels in obtained samples. Haines et al. 1977 enrolled three groups of subjects: 30 patients with CRC (19 with carcinoma of the rectum and 11 with carcinoma of the colon), 64 patients with non-malignant large-bowel disorders, and 208 subjects without known large-bowel disorders. Paired end-expiratory breath-samples were taken using one of two similar methods, by means of either a modified Haldane–Priestley tube or a three-bag collecting system in which one bag contains sample which can then be transferred to a syringe or evacuated aerosol can for later analysis. Determination of methane was carried out using GC. A total of 14 out of 19 patients with rectal carcinoma produced methane, as well as 10 out of 11 patients with colonic carcinoma. In the group of 30 CRC patients, 24 (80%) had significant levels of methane in their breath (mean: 28.8 ± 20.9 ppm), compared with 25 (39%) of 64 patients with non-malignant large-bowel disorders (mean: 16.8 ± 12.7 ppm) and 83 (40%) of 208 subjects without large-bowel affliction (mean: 16.7 ± 13.8 ppm) [44].

Direct gas sampling combined with GC-flame ionization detector (FID) was again used to measure methane concentrations in breath of 270 subjects (Piqué et al. 1984). End-expiratory breath samples were collected using a modification of the Haldane–Priestley tube and stored in 50-mL plastic syringes. Sixty-seven (42.9%) of the 156 healthy controls were CH_4_ producers and forty-three (91.4%) of the 47 patients with CRC were CH_4_ producers (the percentage was significantly higher; *p* < 0.001). In 36 patients in whom the cancer was resected, the incidence of methane producers fell to 47.2%. The percentage of methane producers in patients operated on, but with unresectable cancer, remained very high (87.7%). A significantly higher proportion of patients with extensive ulcerative colitis and colonic polyposis produced CH_4_ than patients suffering ulcerative proctosigmoiditis, benign diseases of the colon, and healthy controls (*p* < 0.05). Values were expressed only as percentage of incidence of methane producers [45].

Peng et al., 2010, employed a tailor-made array of cross-reactive sensors based on organically functionalized gold nanoparticles (GNPs) and HS-SPME-GC-MS to discriminates between breath volatiles of healthy controls (22) and patients suffering different types of cancers, namely lung (30), breast (22), colorectal (26), and prostate (18). Principal component analysis (PCA) demonstrated clear separations between the patterns of healthy controls and of patients suffering from lung, colon, and breast cancers when using the GNP sensors. HS-SPME-GC-MS experiments of breath samples resulted in the finding of six potential biomarkers of colorectal cancer, four with decreased levels (i.e., 1,3-dimethylbenzene, 2-amino-5-isopropyl-8-methyl-1-azulenecarbonitrile) and two increased (1,1′-(1-butenylidene)bis benzene and 1-iodo nonane). The sensitivity of the GC-MS method to colon cancer was about ~30% [46].

Altomare et al., 2013, analyzed breath by means of thermal desorption-gas chromatography-mass spectrometry (TD-GC-MS). The participants included were 37 patients (positive outcome from colonoscopy examination) and 41 controls. Probabilistic neural network (PNN) was used to assess discrimination between cancer and control group and this evaluation provided a model based in a set of 15 compounds. The selected indicators of CRC were: nonanal, 4-methyl-2-pentanone, decanal, 2-methylbutane, 1,2-pentadiene, 2-methylpentane, 3-methylpentane, methylcyclopentane, cyclohexane, methylcyclohexane, 1,3-dimethylbenzene, 4-methyloctane, 1,4-dimethylbenzene, 4-methylundecane and trimethyldecane. The sensitivity was 86% and the specificity was 83%. The AUROC value for this PNN model was 0.852 [47].

Depalma et al., 2014, collected samples of breath from 20 patients with colonoscopic diagnosis of colonic polyps, 15 CRC patients and 15 healthy controls (negative at colonoscopy). They used TD (thermal desorption) combined with GC-MS method to obtain VOC patterns. A linear discriminant analysis (LDA) enabled a discriminant performance with an accuracy of 96.5% and a sensitivity of 100%. The selected model provided correct classification of 14 patients with polyps over 15 for the group of patients with colorectal cancer, with a sensitivity of 93.3%. Group with polyps were distinguished as markedly pathological [48].

Wang et al., 2014, studied breath samples employing SPME. Twenty samples were collected from control group and 20 belonged to individuals with colorectal cancer. The discriminative models were created with the performing of PCA and partial least-squares discriminant analysis (PLSDA). The main potential markers were cyclohexanone, 2,2-dimethyldecane, dodecane, 4-ethyl-1-octyn-3-ol, ethylaniline, cyclooctylmethanol, trans-2-dodecen-1-ol, 3-hydroxy-2,4,4-trimethylpentyl 2-methylpropanoate, in significantly lower levels in positive samples were 6-*t*-butyl-2,2,9,9-tetramethyl-3,5-decadien-7-yne (*p* < 0.05) [49].

TD-GC-MS was employed again by Altomare et al., 2015, who investigated breath from 48 CRC patients and 55 healthy controls. They found 31 VOCs discriminating CRC patients from follow-up (patients after resection with curative intent; FU) groups and from FU groups and healthy groups. The reliability of the calculated PNN model in discriminating between the CRC and the FU groups showed a sensitivity of 100%, a specificity of 95.83%, an accuracy of 97.50%, and an AUC of 0.993. When comparing the FU and healthy control groups with a set of those 31 biomarkers, a sensitivity of 100%, a specificity of 96.36%, an accuracy of 97.70% and an AUC of 0.992 were noted. A total of 11 VOCs were common to the previous study [47] and the PNN analysis using only them, resulted in discrimination of follow-up from CRC patients before surgery with a sensitivity of 100%, a specificity of 97.92%, an accuracy of 98.75%, and an AUC of one. Moreover, the FU group was distinguished from the healthy group which showed a sensitivity of 100%, a specificity of 90.91%, an accuracy of 94.25%, and an AUC of 0.959 [50].

Four compounds were assigned as biomarkers of colorectal cancer by Amal et al., 2016. The amounts of acetone and ethyl acetate were elevated for CRC patients, ethanol and 4-methyl octane were lower for the diseased group. A total of 65 patients with CRC, 22 with advanced or nonadvanced adenomas, and 122 healthy controls were enrolled to the study. Two different techniques were employed for the experiments: sensor analysis with a pattern recognition method and TD-GC-MS. Patients suffering with CRC were distinguished from the control group using a sensor with 85% sensitivity, 94% specificity and 91% accuracy. The advanced adenoma group from the non-advanced adenomas was discriminated with 88% sensitivity, 100% specificity, and 94% accuracy. Finally, the advanced adenoma group from healthy controls was discriminated with 100% sensitivity, 88% specificity, and 94% accuracy. Acetone and ethyl acetate were found in elevated levels in CRC patients (999.6 ± 116.8 ppb and 128.4 ± 4.01 ppb, respectively) compared to the healthy subjects (731.2 ± 63.8 ppb and 41.80 ± 10.00 ppb, respectively). Ethanol and 4-methyl octane were increased in the control group (464.7 ± 61.7 ppb and 19.1 ± 0.8 ppb, respectively) compared with the CRC patients (95.9 ± 48.1 ppb and 16.0 ± 0.63 ppb, respectively) [51].

**Figure 1:**
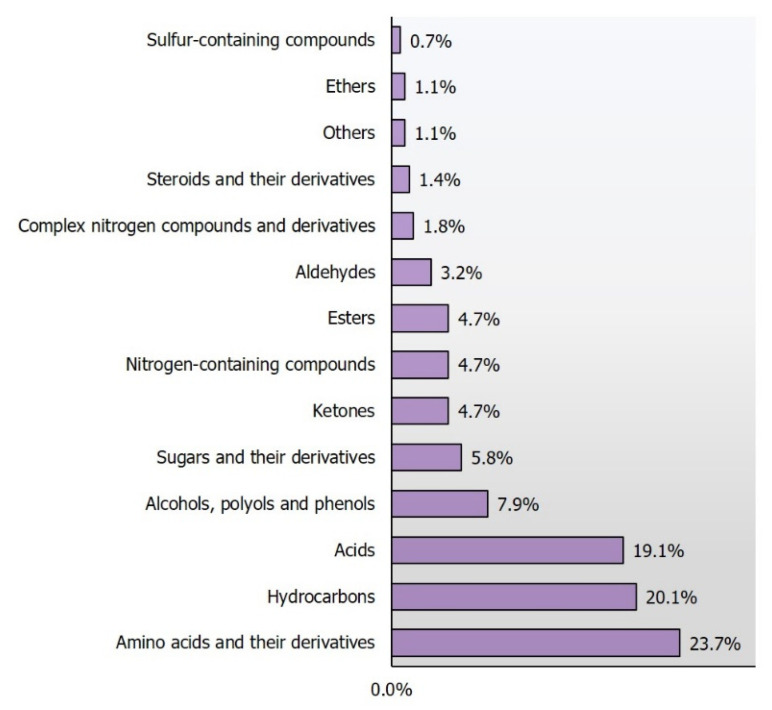
Incidence distribution of potential CRC biomarkers, according to functional group.

In total, 205 different compounds (278, considering substances reported more than once) were indicated as potential CRC markers from all three matrices. Figure 1 and Table 3 show the functional group distribution of compounds in the matrices, divided by matrix. For urine specimen the most prevalent group of compounds were amino acids and their derivatives, acids and sugars with their derivatives. These two first groups were also the most predominant in feces, whereas breath samples contained mainly hydrocarbons. Table 3 demonstrates that 23.7% of all identified potential biomarkers of CRC were amino acids and their derivatives, followed by hydrocarbons (20.1%) and acids (19.1%). The compound with the highest incidence of occurrence among matrices was *p*-cresol (reported by five different studies). The other compounds frequently detected were: hexanal, 2-methylbutane, methylcyclohexane, citric acid, linoleic acid and glutamic acid (all reported at least by four different studies).

### 2.7. Clinical Relevance of Reviewed Articles

Breath, urine and feces can be collected noninvasively from subjects, in contrast to conventionally used matrices (blood, serum, and tissue). Noninvasiveness is an important factor for patient safety and may reflect on increased rates of adherence to the test, helping to promote early diagnosis. Besides that, simplified sample collection may not require qualified personnel. The mentioned biological samples are specimens which can be taken relatively fast, at little cost, and without extensive sample preparation for analytical instruments. The unfavorable issues concerning urine and feces are the possible discomfort experienced by patients and the complex chemical nature of them. Although breath has lower chemical complexity, various external factors can influence its composition, such as food remnants, hygiene products, metabolic products of colonizing oral bacteria and ambient air [57,58,59,60], turning challenging to address which fraction of it would represent blood-related biomarkers.

Urine is a more stable specimen and contains mainly water, inorganic salts and organic compounds, such as hormones, proteins, other metabolites and bacteria with their products. Due to the role of urine as organism’s route of elimination, metabolized forms of substances prevail over parental molecules. Regarding bioanalyses of urine, there are a few recommendations intended to avoid excessive contribution of bacterial content in the samples. In fact, the influence of bacterial growth on the levels of metabolites and proteins has been reported [61]. It was demonstrated that the mid-stream portion of urine has reduced microbiota composition [62]. Time collection period and diet are also significant parameters; in this sense, fasting urine was characterized as the most stable in terms of basal composition [63]. The determination of urine specific gravity is an alternative for normalization of samples for metabolic investigations [64]. Most of the studied urine samples in this review were collected in the morning after overnight fasting [31,32,33,35]. Further information regarding detailed protocol of sampling was not provided.

Feces are an especially meaningful biological specimen in what concerns the evaluation of colonic system. These are typically solid or semisolid heterogeneous remains of food, not completely digested or absorbed by the organism. This content is metabolized by intestine bacteria to smaller waste products and can also contain dead epithelial cells from the lining of the gut. Diet can change the composition of feces, which directly alter metabolite profiles [65]. Moreover, consumed food impacts metabolic pathways of gut microbiota and volatiles produced by them [66]. Factors related to the collection and handling of fecal samples like post-collection metabolite deterioration, due to exposure to aerobic conditions and ongoing microbial fermentation, also take place [67]. Investigations of fecal samples regarding VOC profiles, revealed no effect when comparing a processed fresh sample (firstly homogenized and extracted) to a thawed sample after 7 days, kept at −20 °C [68]. Interestingly, lyophilized feces showed a decrease in the number of detected analytes [69].

All revised studies regarding fecal samples did not impose any kind of dietary restrictions to the patients. In certain studies, samples from individuals following vegetarian diet or having any dietary restrictions were not included [38,43], as this factor itself could reflect on a differentiated profile of molecules extracted from the collected specimen. Interruption of consumption of tobacco or alcohol was also required in a study [41]. Song et al., 2018, collected samples provided after overnight fasting and participants were instructed to consume solely local traditional food [42]; participants enrolled in another study all had a mild diet [41]. Fecal samples were generally provided before the colonoscopy, without the influence of any kind of procedure for bowel preparation [38,41,42,43]. Another common exclusion criteria regards the intake of antibiotics and probiotics months prior to sample donation, once these agents impact the natural composition of gut microbiota [41,43]. In most of the studies, patients suffering from intestinal chronic inflammatory diseases were also not enrolled [38,39,41,43], due to the possibility that the metabolic alterations promoted by these conditions may result in a false positive or a confounding factor when CRC cases are evaluated. The composition of feces, urine and breath can also vary according to the physical state of the body, age, and general health. Hence, biomarkers found in these matrices can have multifarious origin that should be considered while establishing potential metabolic pathways of them [59,70,71].

The revised studies can be critically analyzed under the point of view of their clinical value. In urine metabolome studies, Qiu et al., 2010, indicated separations both from CRC patients and precancerous colorectal lesions in rats to their healthy analogous counterparts. It is an interesting result because indication of precancerous lesion is the main limitation of commercially available CRC tests which do not provide good sensitivity in the first stage of the disease. The main differences observed between pre- and post- groups would be due to removal of a portion of the microbiota during preoperative preparation of the colon and the influence of supplements intake. These authors suggested that general results point mainly to changes in tryptophan metabolism and tricarboxylic acid (TCA) cycle due to the CRC development. Moreover, the method’s reliability was corroborated since almost half of the altered substances had their identity confirmed by the analysis of standards [31]. Cheng et al., 2012, similarly to Qiu et al., 2010, suggested the tricarboxylic acid (TCA) cycle, tryptophan metabolism, and polyamine metabolism as the main affected pathways in the CRC metabolome. Additionally, they achieved receiver operating characteristic (ROC) curves indicating a method accuracy close to 100% [33]. Liesenfeld et al., 2015, monitored the metabolomes of patients over time, in the frame of the ColoCare project—a study encompassing a series of interventions for the collection of samples and data at different time-points during the period of 5 years. A clear distinction was presented between the metabolisms of pre- and post- operative cohorts. Their result supported the hypothesis that the gut microbiota play a more important role for colon cancer than for rectal or rectosigmoidal cancer patients. Removal of cancerous tissue and parts of the intestine may affect microbial metabolites and possibly the microbiota itself. The increased effect is intensified with adjuvant chemotherapy. Once again, subjects’ metabolism was evidenced to be affected by changes in the levels of tryptophan. One limitation of the study was impossibility to identify some metabolites using the available spectral library [35]. Primarily, the study of Delphan et al., 2018, was focused on determination of branched-chain amino acids (BCAAs). However, no correlation between the amounts of BCAAs and some evaluated energy balance parameters were observed. On the other hand, two acids were significantly elevated in the CRC group and associated with overall survival [36]. Silva et al., 2011, were able to discriminate colorectal cancer from other types of neoplasm (leukemia and lymphoma). In this case, authors presented data regarding the optimization of the sample preparation method for the selection of parameters with the greatest recovery of compounds [32]. GC-MS was used as complementary technique by Arasaradnam et al., 2014, in which an ITEX device served in the preconcentration step. ITEX is a solution that provides sensitivity similar to purge and trap systems and requires less instrumental effort, with lower susceptibility to contamination [34,72]. Usage of two employed methods (FAIMS and GC-IMS) from the described protocol, did not provide satisfactory differentiation between the adenoma and healthy groups, however, singular CRC cases could be correctly classified from control and adenomas using the mentioned techniques. The authors highlighted that a panel of biomarkers is preferential for differentiation, rather than using a single biomarker for diagnostic purposes. This study was focused on individuals that tested positive for FOBT, then, the results were compared with actual diagnostic outcomes. In this sense, the proposed metabolome-based methodology proved to be superior compared to the FOBT approach [37].

As mentioned before, bacterial content strongly influences the composition and metabolic outcome of fecal samples. Weir et al., 2013, investigated stool samples from a small group of CRC and healthy patients. The authors stated that using sequencing, allied together with metabolic profiles may be a powerful approach in the elucidation of mechanisms of interaction between gut microorganisms and metabolites. They quoted the hypothesis of the driver–passenger model, in which bacteria are infectious agents in the development of cancer. Bacterial drivers of colorectal neoplasm are intestinal bacteria with pro-carcinogenic features. One of these features is the production of DNA-damaging compounds. ‘‘Passenger bacteria’’ are bacteria that may outcompete drivers to flourish in the tumor environment as the cancer progresses. The findings from Weir et al., 2013, lead to the conclusion that by knowing the relationship between colonizing bacteria and altered fecal metabolites, there is the possibility to focus on metabolome analyses to indirectly assess the composition of microbiota, and if the latter implies CRC risk [38,73]. Phua et al., 2014, recognized that the used sample set was rather small for the aimed purposes. They mentioned that a previous study regarding fecal samples (Weir et al., 2013) was potentially affected for not considering the presence of blood in the stool samples, making difficult to determine whether the found metabolites would be originated from blood or not. With respect to this, in their study the authors worked with a subset of stool samples without a noticeable amount of blood, aiming to mitigate the interferences of this matrix on the conducted experiments [39]. Wang et al., 2017, also focused on the bacterial content of feces and its relationship to secreted metabolites. They signalized that for a better assessment of the correlation between bacterial genus and detected compounds, a larger group of samples should be considered [41]. In contrast to other researchers, Song et al., 2018, pointed out that no significant differences were found regarding the levels of fecal short-chain fatty acids in CRC samples. Through the monitoring of the diet of a healthy group, it was demonstrated that there is no correlation between described dietary habits and level of substances of interest, suggesting that other factors (specifically related to CRC) contribute to the differential distribution of fatty acids in feces [42]. Bond et al., 2019, hypothesized that propan-2-ol is a major metabolite of *Fusobacterium nucleatum*, a strain previously connected with CRC tumorigenesis. Despite that main candidate biomarkers were identified by analysis of chemical standards, no quantification was performed [43].

The earliest works comprising exhaled breath from CRC patients concerned the detection of methane. The study of Haines et al. 1977, was focused on the evaluation of this gas and encompassed the collection of the last portion of patients’ expiration. Methane levels in the room air were taken as a basal measure to define methane producers and non-producers. Excluding univariate comparison between the two groups, no further statistical analyses were explored. The results revealed poor sensitivity and specificity, reinforcing that approaches relying on the determination of a single marker tend to be deficient; however, the used approaches offered perspectives towards CRC breath diagnosis. Nowadays, different options of analytical apparatus are available, able to provide increased sensitivity and detection of a wider range of compounds from different classes. Considering the limitations inherent to the period, this work was the first to suggest the evaluation of bacterial metabolites as a manner to assess the presence of CRC tissue [44]. Due to the coincident prevalence of methane in the studied cohorts, results of Piqué et al. 1984, suggested the existence of a common substrate for large bowel cancer, extensive ulcerative colitis, and colonic polyposis. Produced metabolites found in the breath from CRC patients are likely to originate from modified microflora [45]. In the work of Peng et al., 2010, the main goal was to enable differentiation of various types of cancers using nanosensors, since GC-MS was used as validation tool. The employed methodology did not perform so satisfactorily for CRC, in comparison to other studied neoplasms—it was pointed out that a significant portion of the CRC profile was overlapping with VOC patterns coming from healthy subjects. This may indicate that sensed CRC metabolic changes were rather subtle [46]. Altomare et al., 2013, used for the first time TD-GC-MS in such context. The main advantage of the thermal desorption technique using sorbent cartridges, is the efficient concentration of analytes, allowing VOCs trace analysis without requiring any sample preparation steps [74,75]. The researchers used a sample collection protocol based on the vital capacity expiration instead of prioritizing obtaining alveolar breath. This procedure can increase the influence of exogenous compounds in breath composition, however, it is a more comfortable and reachable approach for patients [47]. Depalma et al., 2014, were able to differentiate between patients with polyps and patients with CRC. Once the majority of CRC cases derive from polyps, the detection of such markers (as achieved by the referred study) is significantly relevant. Polyps’ detection may indicate which individuals should be monitored regularly in order to prevent disease development [48]. In the work of Wang et al., 2014, the differentiation between tumor stages was studied, however, no specific patterns could be associated with the varied degree of tumor growth. The researchers used a collection procedure based on obtaining alveolar breath samples, thus favoring the evaluation of compounds more closely related to blood levels (alveoli–blood exchange), which suffers less influence from room air composition [49]. Altomare et al., 2015, showed for the first time that breath profiles are also subjected to differences when comparing patterns from positive groups with the same cohort after undergoing curative tumor resection. In addition, they highlighted the need for more complex chemometric models to achieve the identification of specific patterns, leading to a successful breath test [50]. GC-MS was used in a study by Amal et al., 2016, to identify candidate biomarkers related to the results of sensor analyses. Authors performed quantitation of detected potential biomarkers, however, details of the calibration procedure were not demonstrated in the article [51].

## 3. Possible Origin of Potential Molecular Biomarkers of CRC

There are several complex mechanisms involved in the carcinogenesis process, which comprehend changes in cell biochemistry and a series of metabolic adaptations, characteristic of tumor development. Most of these mechanisms are still not fully elucidated and their investigation at the level of small metabolites remains insufficient. Considering this, the present section aims to discuss the main hypotheses related to the occurrence and modulation of the compounds most frequently reported as potential CRC biomarkers. For this purpose, aforementioned candidate markers of CRC were correlated with molecular pathways previously described as related to this disease. Solely mechanisms which could be addressed to the altered metabolites reported in the previous section were presented. Through the next section, references are made to the observations presented in Table 2.

### 3.1. Alterations in Cell Energetics

During tumorigenesis, the cells demand the reprogramming of energy generation. Apart from the favoring of glycolysis (Warburg effect) [76,77,78], perturbations of the TCA cycle are also documented. In the TCA cycle, intermediates which can be incorporated in biosynthetic pathways are formed; therefore, this cycle can be found to be partially down-regulated, due to the need of malignant cells for precursors [78,79]. Apart from this, alterations in genes related to the expression of TCA cycle enzymes are reported [80]. The beta-oxidation of fatty acids seems to be triggered by the aforementioned metabolic disturbances. It is demonstrated that this process can be parallelly stimulated to serve as another energy sustaining source, once fatty acids are broken down to give rise to acetyl-CoA, ATP (adenosine triphosphate) and reduced co-enzymes [81].

In most of the studies related to CRC, lactic acid (M54) is found to be increased in biological matrices, such circumstance can be due to the enhanced secretion of this metabolite during the upregulated glycolysis in oxygen-deprived environment [82]. Partial inactivation of the TCA cycle is evidenced in CRC, with its corresponding intermediates citric, isocitric, succinic and fumaric acids (M47, M51, M61, M63, respectively) presenting decreasing trends in comparison to urine control samples.

Acetic acid (M44) was pointed out as a potential cancer indicator, according to the investigation in fecal samples belonging to individuals with cancer. Acetate is mostly sourced from intestinal fermentation performed by bacteria; however, current research verified that acetate could also be produced from pyruvate (yielded at the end of glycolysis), this conversion would be promoted by reactive oxygen species (ROS) [83,84]. The assimilated acetate can be converted to acetyl-CoA and may configure as a substantial energy source in a poor nutrient environment and under hypoxia conditions, which are characteristic of tumors [84,85].

### 3.2. Structural Self-Maintenance

The glycerol produced in the cytosol can be phosphorylated and submitted to consecutive reactions, covering the generation of phospholipids and glycerides. In this sense, the glycerolipid pathway comprises a source of building blocks required for cell survival and growth; concurrently, produced lipids have a function as signaling molecules with pro-tumorigenic properties in cancer [86,87]. When compared to control fecal samples, glycerol (M6) was found in lower abundance. Apart from this, monoacylglycerol (M7), an intermediate of the same pathway, also presented lower responses in the positive cohort. These observations can be connected with the greater consumption of glycerol in CRC, due to the enhancement of glycerolipid biosynthesis.

**Figure 2:**
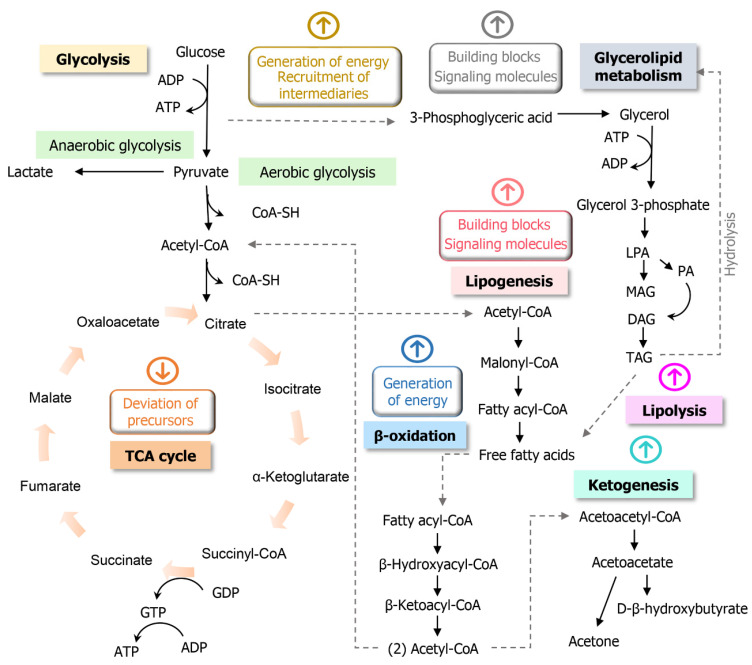
Connected main pathways possibly altered in CRC (related to the reported metabolic biomarkers), involved in energy production and the generation of functional biomolecules, where: TCA—tricarboxylic acid, ATP—adenosine triphosphate, ADP—adenosine diphosphate, GDP—guanosine diphosphate, GTP—guanosine triphosphate, LPA—lysophosphatidic acid, PA—phosphatidic acids, MAG—monoacylglycerol, DAG—diacylglycerol, TAG—triacylglycerol. Based on Anderson et al., 2018 [79], Icard et al., 2012 [81] and Prentki et al., 2008 [87].

Lipogenesis also appears upregulated in cancers, generating material for cell membrane building and saturation, and the biosynthesis of lipid-signaling molecules [88,89]. Additionally, the lipolytic pathway seems to be explored by cancer cells as a manner of using exogenous fatty acids to enhance their own growth [89]. Considering this context, species related to the lipolysis pathway are also expected to exhibit alterations. Linoleic (M55) and oleic acids (M57), the main constituents of cell membranes, are recurrently reported as decreased in fecal samples of CRC individuals, an observation coincident with the withdrawal of fatty acids. Extending this analysis and considering the occurrence of an excessive breakdown of fatty acids, the greater occurrence of ketone bodies can be supposed. Both 3-Hydroxybutanoic acid (M53) and acetone (M21) were found with augmented levels in the urine and breath of CRC patients, both compounds are ketone bodies derived from acetoacetate decarboxylation occurring in the final stage of lipolysis pathway. Figure 2 presents a scheme of the main pathways possibly altered in CRC.

During carcinogenesis, a series of molecular signals culminate in the activation of the transcription of genes linked to the mevalonate pathway, causing dysfunctions in the levels of intermediate metabolites. This would be a mechanism of tumor cells to restore the levels of molecules that are products of this pathway, which have important structural functions [90,91]. Terpenoids indicated as cancer biomarkers can have their origin related to unrevealed mechanisms laying on aberrant activity of mevalonate pathway or even represent intermediates from these pathways that were not properly identified by the spectral library. Besides this, the differentiated occurrence of such compounds could be associated with deficient processes in the metabolization of secondary plant metabolites coming from the diet. p-Cymene (M41) and γ-terpinene (M42) were terpenoids found increased in CRC urine specimens, and beta-pinene (M43) in breath samples.

### 3.3. Oxidative Stress

The concentration of ROS is often reported to be elevated in tumors—these species can be produced in the cell as consequence of enhanced metabolic activity and mitochondrial damage, but also play important role in cancer signaling pathways [92,93].

**Figure 3:**
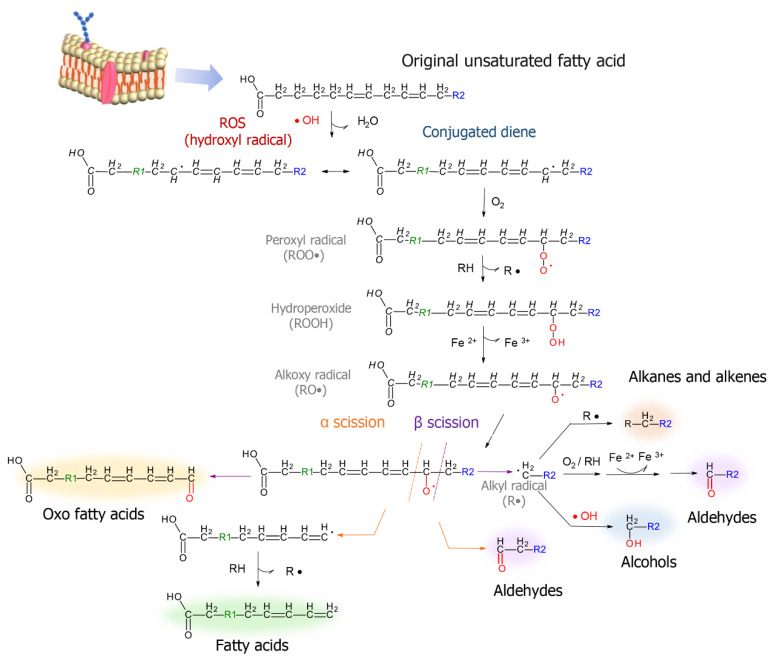
Lipid peroxidation mechanisms potentially involved in the formation of main classes of metabolic biomarkers. Based on Miekisch et al., 2004 [94] and Schaich et al., 2013 [95].

Oxidative stress also favors lipid peroxidation, generating products such as linear alkanes, aldehydes and alcohols (Figure 3) [94]. Nonanal (M15) and decanal (M12) were present in greater amounts in breath of CRC patients, these are compounds that can be directly associated with the oxidation of the main lipids constituting cell membrane. Nonanal can be produced both by α and β-scissions, while decanal may arise from the β-fragmentation of oleic acid [95]. Alkanes such as dodecane (M37), 2-methylbutane (M30), 2-methylpentane (M31), 3-methylpentane (M32), 4-methyloctane (M35)—also elevated in exhaled air of positive cohort, are likely other products of lipid oxidation that have not been precisely described yet.

### 3.4. Alterations in Enzyme Catalytic Activity

The activity of cytochrome P450 is reported to be altered in different neoplasms, through the differential expression of their isoforms. This factor can influence the bioavailability of substrates and their products, as well as their pattern of excretion in cancer cases [96,97]. Alcohol dehydrogenase and aldehyde dehydrogenase present activities statistically superior in several tumors when compared to normal tissues [98]. It is reported that reduced co-enzymes produced by the action of these enzymes may support ATP production [99].

Magnified activity of aldehyde dehydrogenase in tumors can transform the aldehydes released during lipid peroxidation into corresponding carboxylic acids. In this sense, secondary ketones may arise from carboxylic acids undergoing β-oxidation process—a mechanism boosted in cancer cells, as previously mentioned. Such observations regarding metabolic dynamics in cancer may explain why n-aldehydes heptanal (M13) and hexanal (M14) were decreased in the urine of individuals belonging to the positive group, while the ketones 4-heptanone (M18) and 2-pentanone (M20) presented a greater response in urine samples from the diseased. Because urine consists of a fluid related to the elimination route in organisms, the assumption that molecules are in their metabolized form may be reasonable. Compounds such as cyclohexane (M36), methylcyclohexane (M39) and xylene (M27, M28) in the breath were pointed out as CRC markers. These compounds are traditionally addressed as exogenous, coming from environment. The endogenous synthesis of such VOCs was not described in humans yet, nevertheless, bacterial shikimate and derived pathways could play a part in the occurrence of similar aromatic compounds. Another hypothesis is that altered isoforms of enzymes responsible for the metabolism of these exogenous compounds are impaired during cancer, making increased levels of these cyclic and aromatic VOCs frequently observed among diseased patients.

### 3.5. Contribution of the Microbiota

One of the most important roles of intestinal bacteria is the fermentation of saccharides that cannot be digested by human. Dietary fibers can exhibit different levels of susceptibility to the fermentation process and, when low fermentable, appear to be related to colonic diseases [100]. In bacterial fermentation, released monosaccharides are converted into pyruvate and derived short-chain fatty acids are produced—from these, the major species formed are acetate, butyrate and propionate [100,101]. In CRC collected fecal samples, acetic (M44) and propionic acid (M59) were registered with augmented responses. The indexed literature reports butyric acid (M46) as a discriminant feature in CRC; however, diverging trends in butyric acid levels are documented.

**Figure 4:**
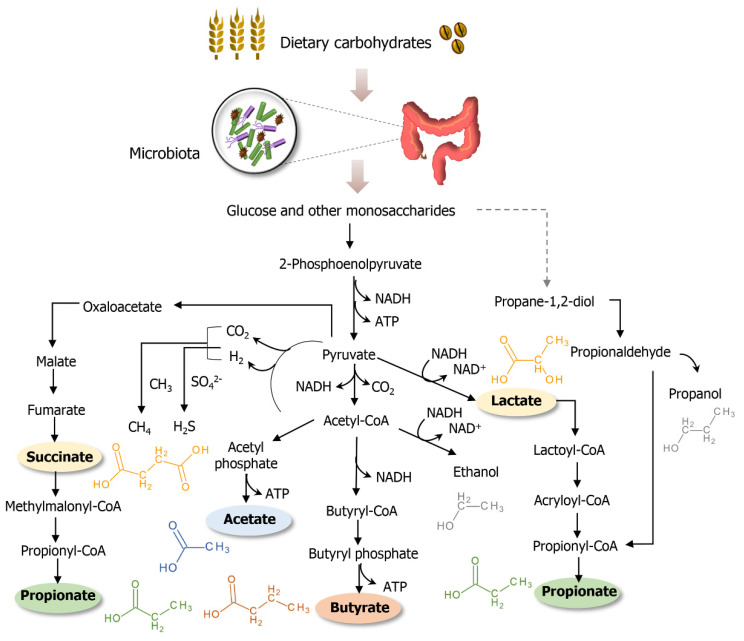
Bacterial fermentation pathways in the human gut. Based on Commane et al., 2005 [102] and Koh et al., 2016 [105].

The minor metabolites of the fermentation pathways are methane, hydrogen sulfide, ethanol and formate [102]. Methane (M25) was reported with higher concentrations in the breath of colon cancer subjects. In intestinal gas samples, the hydrogen sulfide concentration was elevated, while its precursor—hydrogen—was decreased [103]. Ethanol (M5) measured in exhaled air presented to be distinguished for healthy and diseased subjects. Succinate (M61), a propionate precursor, was present in urine at lower abundance; correspondingly, propionic acid (M59) itself was detected in superior amounts in feces. Fermenting microorganisms can convert acetone to 2-propanol (M3) [104], a compound found to be increased in fecal samples in CRC cases. A scheme of microbial fermentation pathways in the gut environment is depicted in Figure 4.

An alternative fermentation pathway through acetoin metabolism may be performed by bacteria. This path involves the formation of 2,3-butadione (M22) and 2,3-butanediol (M10) [106,107]. The production of 2,3-butanedione is believed to be supported by an acidic pH and low oxygen environment, conditions compatible with the tumor micro-region. This substance appeared in elevated amounts in urine samples of CRC subjects.

Based on the exposed, and according with the reviewed studies, an accumulation of some short-chain fatty acids in CRC is evidenced. In fact, it is demonstrated that butyrate (M46) has its oxidation reduced in CRC tissue, the process is achieved through the down-regulated expression of short chain acyl-CoA dehydrogenase [108,109]. Specifically, butyrate promotes the development of normal cells and inhibits histone deacetylases, thus, making it reasonable to assume that malignant colonocytes perform a metabolic shift to skip butyrate as an energy source [109].

Mucins are glycosylated proteins frequently overexpressed by different epithelial cancer cells. They play a role in the control of the inflammatory response and it is believed that their molecular apparat is employed by tumor cells to stimulate cell growth and survival [110]. In parallel, it is demonstrated that the cancer environment can favor bacterial genera that perform mucin degradation in the intestine [111,112]. Lower levels of sugars and derivatives (M90-99) in feces and urine from CRC patients are indicative of the recruitment of these species for fermentation. Apart from this, free amino acids (M73, M74, M76, M79, M80, M82, M83, M85, M86, M89) were encountered as being elevated in fecal samples, suggesting degradation of mucins and other proteins in the colon.

**Figure 5:**
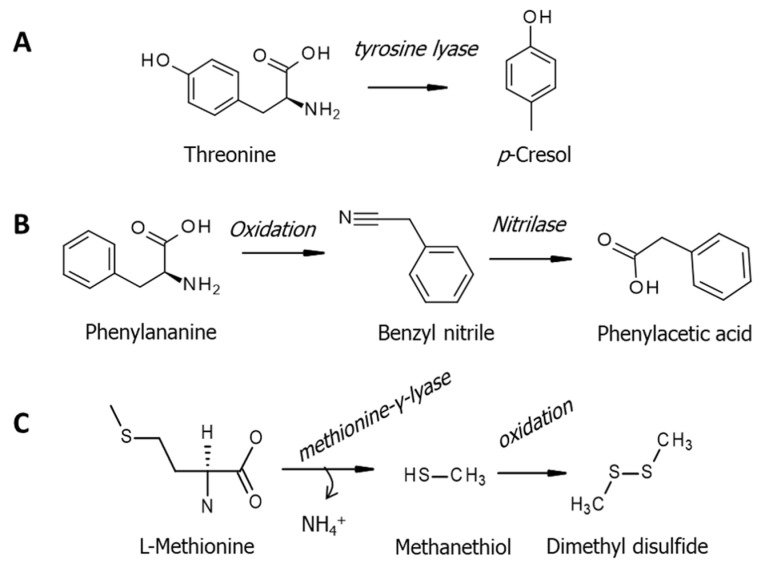
Amino acid metabolism addressed to bacteria: (**A**) threonine degradation; (**B**) aldoxime-nitrile pathway and (**C**) methionine catabolism. Based on Portune et al., 2016 [115], Bhalla et al., 2018 [116] and Furne et al., 2001 [117].

The microbial catabolism of proteins in the gut is another factor to be considered [113]. Proteolytic fermentation is considered as non-favorable, once reactive products can be generated, leading to tissue inflammation, a process that can serve both to initiate and promote colorectal cancer [114]. Neoplastic lesions appear to be induced by the formation of detrimental amino acid metabolites, such as *p*-cresol (M4) [115] (Figure 5A). Similarly to *p*-cresol, phenyl acetate (M23) was also encountered with elevated amounts in the urine of CRC patients. The later can be derived, for example, from phenylalanine catabolism by the microbial aldoxime–nitrile pathway [116] (Figure 5B).

Hydrogen sulfide and methanethiol are harmful species produced in the bowel by the reduction of sulphate-derived substrates [117,118] (Figure 5C). Stimulated production of sulfur-containing species has been addressed in colorectal cancer, as an indicator of alterations in gut bacteria activity driven by carcinogenesis [118]. Potentially related to this, the levels of dimethyl disulfide (M65) and 2-methoxythiophene (M64) appeared altered in positive urine samples.

Elevated amounts of free amino acids in feces—a colon directly related sample—can be also indicative of poor nutrient fixation in the previous steps of digestion. The observed increment in the concentration of the aforementioned metabolites in CRC specimens demonstrates that CRC-associated gut microbiota actively perform amino acid catabolism, which corroborates to colon inflammation and may constitute a tumor promoting mechanism by triggering an inflammatory response which activates cell division.

## 4. Conclusions

Considering the reviewed articles, it is possible to observe that varied methodologies have been employed for the study of the CRC metabolome. The investigation of different biological species has permitted observations of varied aspects of changes in metabolism, as each matrix involves different physiological mechanisms. Chemometric approaches were prevalent among the reviewed studies, presenting themselves as indispensable for the processing of the complex data acquired. Such statistical evaluations enable us to identify discriminant features and latent patterns, as well as to express the method’s performance. The most prevalent altered compounds observed in the investigated profiles were hydrocarbons, short-chain fatty acids and amino acids and their derivatives. These chemical species could be correlated with general cancer mechanisms and specific pathways affected during CRC. The indexed candidate biomarkers could be addressed as metabolites, both of human and bacteria from the gut microbiota. The inspected studies indicate the promising status of the analysis of small molecules, using non-invasive approaches, in the determination of CRC with an accuracy greater than the already available screening tests. Besides that, GC-based exams have great potential to configure as affordable and standardized methods, able to attend to broad demand. Nowadays, there is an expanding group of evidence showing metabolite-based diagnosis of CRC, however, data inconsistency due to the employment of diverse protocols is observed, emphasizing the need for validation strategies. Yet, the value of independent studies cannot be diminished, for the reason that the gathered evidence presents great elucidative value in what concerns the understanding of particular mechanisms associated with CRC. In this way, studies on molecular patterns can be applied for diagnostic purposes, as well as to configure as a powerful tool for the interpretation of disease mechanisms at the molecular level.

## Figures and Tables

**Table 1:** Table summarizing all 21 studies regarding investigation of biomarkers of CRC in urine, feces, and breath samples.

Reference	Subjects	Sample Preparation and Analytical Technique	Main Analytes	Type of GC Column	Statistical Approach
**URINE SAMPLES**					
Qiu et al., 2010 [31]	60 CRC: ➢ stage I: 7 ➢ stage II: 23 ➢ stage III: 21 ➢ stage IV: 9 63 HC	solvent extraction with chloroform and derivatization with ECF + GC-MS	SNM: amino acids; organic acids	DB-5MS capillary column (30 m × 250 µm i.d., 0.25-μm film thickness)	PCA, OPLS-DA
Silva et al., 2011 [32]	12 CRC HC	HS-SPME with CAR/PDMS (75 µm) + GC-MS	SVM: hydrocarbons; aldehydes; sulfur compounds	30 m × 0.25 mm ID × 0.25 µm film thickness BP-20	one-way ANOVA, LSD, PCA
Cheng et al., 2012 [33]	103 CRC: ➢ stage I: 24 ➢ stage II: 45 ➢ stage III: 27 ➢ stage IV: 5 101 HC	solvent extraction with methanol and derivatization with methoxyamine (in pyridine) and BSTFA (1% TMCS) + GC-TOFMS	SNM: amino acids; organic acids; saccharides	DB-5MS capillary column (30 m × 250 µm I.D., 0.25-μm film thickness; (5%-phenyl) methyl-polysiloxane bonded and cross-linked	PCA, OPLS-DA, ROC curve, Student’s *t*-test, Wilcoxon−Mann−Whitney test
Arasaradnam et al., 2014 [34]	83 CRC 50 HC	ITEX + GC-MS	SVM: ketones; aldehydes; nitrogen compounds	Rxi-624Sil column (20 m length, 0.18 mm ID, 1.0 µm df)	FDA, KNN method
Liesenfeld et al., 2015 [35]	Total for GC-MS and ^1^H-NMR is 199 CRC: CRC pre-surgery: ➢ s0: 5; sI: 12; sII: 40; sIII: 22; sIV: 18 CRC post-surgery: ➢ sI: 4; sII: 4; sIII: 2; sIV: 2 CRC 6 months follow-up: ➢ sI: 12; sII: 17; sIII: 15; sIV: 8 CRC 12 months follow-up: ➢ sI: 7; sII: 13; sIII: 14; sIV: 4	solvent extraction with methanol and derivatization with methoxyamine (in pyridine) and BSTFA (1% TMCS) + GC-MS	SNM: alcohols; amino acids; organic acids; saccharides	HP-5 MS fused silica column (30 m × 0.25 mm; 0.25 µm film thickness of the 5% phenyl 95% dimethylpolysiloxane stationary phase	Wilcoxon–Mann–Whitney tests, PLS-DA, one-way ANOVA, ROC curve
Delphan et al., 2018 [36]	163 CRC pre-surgery: ➢ stage I/II: 76; stage III/IV: 87 83 with 6 months follow-up: ➢ stage I/II: 36; stage III/IV: 47 54 with 12 months follow-up: ➢ stage I/II: 32; stage III/IV: 25	solvent extraction with methanol and derivatization with methoxyamine (in pyridine) and BSTFA (1% TMCS) + GC-MS	SNM: amino acids	HP-5 MS fused silica column (30 m × 0.25 mm; 0.25 µm film thickness of the 5% phenyl 95% dimethylpolysiloxane stationary phase	one-way ANOVA, Pearson Chi-squared test, Pearson’s partial correlation coefficients, Cox proportional hazard models
Mozdiak et al., 2019 [37]	12 CRC 80 adenoma 14 diverticular disease 5 haemorrhoids 14 inflammatory bowel disease 1 excluded 37 HC	not specified + GC-IMS	undetermined	not specified	ROC curve, Sparse logistic regression, Random Forest, Gaussian process classifier, Support vector machine, Neural network
**FECAL SAMPLES**					
Weir et al., 2013 [38]	10 CRC 11 HC	solvent extraction with isopropanol:acetonitrile:water and derivatization with methoxyamine (in pyridine) and MSTFA (1% TMCS) + GC-MS	SNM: amino acids; organic acids; lipids; steroids	TG-5MS column (30 m, 0.25 mm i.d., 0.25 µm film thickness), SCFA determination: TG-WAX-A column (30 m, 0.25 mm ID, 0.25 µm film thickness)	AMOVA, Student’ *t* test, ANOVA, Pearson correlation, PLS-DA
Phua et al., 2014 [39]	11 CRC: ➢ sB: 6; sC: 5 10 HC	solvent extraction with methanol:water and derivatization with methoxyamine (in pyridine) and MSTFA (1% TMCS) + GC-TOFMS	SNM: lipids; saccharides	DB-1 (30 min × 250 µm i.d.) fused silica capillary column with 0.25 µm film thickness	PCA, OPLS-DA, ROC curve, Welch *t* test
Bond et al., 2016 [40]	21 CRC 56 with adenomatous polyp/s 60 HC	HS-SPME + GC-MS	SVM	not specified	Student’s *t* test, Fisher’s exact test, ANOVA, false discovery rate correction, PLS-DA, factor analysis, ROC curve
Wang et al., 2017 [41]	15 CRC: ➢ sII: 4; sIII: 6; sIV: 5 12 HC	solvent extraction with isopropanol:acetonitrile:water and derivatization with pyridine-methoxy amino acid salt solution, SCFA determination: solvent extraction and derivatization with sulfuric acid solution (50%) and diethyl ether + GC-MS	SNM: amino acids; organic acids; lipids; steroids	30-m TG-5MS column	Student’s *t*-test, Pearson correlation
Song et al., 2018 [42]	26 CRC: ➢ sI: 3; sIIa: 5; sIIc: 1; sIIIb: 11; sIIIc: 3; sIVa: 3 28 HC	Analysis of Long-Chain Fatty Acids: solvent extraction with chloroform:methanol (Folch method) and derivatization with BCl_3_–MeOH Analysis of Short-Chain Fatty Acids: solvent extraction with HCl and diethyl ether and derivatization with PFBB in acetonitrile and EDIPA + GC-MS	lipids	HP-5 MS 30 m × 250 µm × 0.25 µm column	Chi-square test, Fisher’s exact test, Mann–Whitney *U* test
Bond et al., 2019 [43]	21 CRC 56 with adenomatous polyp/s 60 HC	HS-SPME with CAR/PDMS + GC-MS	SVM: esters; alcohols	60 m long Zebron ZB-624 capillary column with an inner diameter of 0.25 mm. The column was lined with a 1.4 µm film of 94% dimethyl polysiloxane and 6% cyanopropylphenyl	Student’s *t* test, Mann-Whitey tests, Fisher’s exact test, ANOVA, false discovery rate correction, PLS-DA, factor analysis, ROC curve
**BREATH SAMPLES**					
Haines et al. 1977 [44]	30 CRC 64 with non-malignant large-bowel disorders 208 without known large-bowel disorders	direct gas sampling by means of: either a modified Haldane–Priestley tube’ or a 3-bag collecting system in which one bag contains sample which can then be transferred to a syringe or evacuated aerosol can for later analysis + GC	gases	not specified	*p* value
Piqué et al. 1984 [45]	47 CRC 156 HC	direct gas sampling by means of a 3-bag collecting system in which one bag contains sample which can then be transferred to a syringe or evacuated aerosol can for later analysis + GC-FID	gases	not specified	*p* value
Peng et al., 2010 [46]	26 CRC: ➢ sI: 3; sII: 7; sIII: 7; sIV: 7 22 HC	HS-SPME with PDMS/DVB + GC-MS	SVM: hydrocarbons	H5-5MS 5% phenyl methyl siloxane (30 m length, 0.25 mm i.d., 0.25 µm thickness)	PCA
Altomare et al., 2013 [47]	37 CRC 41 HC	adsorption of VOCs on to sorbent cartridges and thermal desorption + GC-MS	SVM: hydrocarbons	SUPELCOWAX, polyethylene glycol 30 m × 0.25 mm ID. × 0.25 µm stationary phase thickness	PNN, ROC curve
Depalma et al., 2014 [48]	15 CRC 20 with colonoscopic diagnosis of colonic polyps 15 HC	adsorption of VOCs on to sorbent cartridges and thermal desorption + GC-MS	undetermined	not specified	LDA
Wang et al., 2014 [49]	20 CRC 20 HC	HS-SPME with CAR/PDMS (75 µm) + GC-MS	SVM: alcohols; hydrocarbons	DB-5MS (length 30 m × inner diameter (ID) 0.250 mm × film thickness 0.25 µm)	PCA, PLS-DA, Kruskal–Wallis rank sum test
Altomare et al., 2015 [50]	48 CRC 55 HC	adsorption of VOCs on to sorbent cartridges and thermal desorption + GC-MS	SVM: hydrocarbons	HP-5MS, 95% polydimethylsiloxane, 5% polydiphenylsiloxane, 30 m × 0.25 mm ID, 0.25 µm stationary phase thickness	Mann–Whitney *U* test, chi-square test, Student’s *t* test, PNN, ROC curve
Amal et al., 2016 [51]	65 CRC 22 with advanced or nonadvanced adenomas 122 HC	adsorption of VOCs on to sorbent cartridges and thermal desorption + GC-MS	SVM: hydrocarbons; ketones; esters; alcohols	SLB-5ms capillary column (with 5% phenyl methyl siloxane; 30 m length; 0.25 mm internal diameter; 0.5 µm thicknesses)	Student’s *t* test, DFA, ROC curve

VOC—volatile organic compound; CRC—colorectal cancer; HC—healthy controls; s—stage of cancer; ITEX—in-tube extraction; ^1^H-NMR—proton nuclear magnetic resonance; GC-MS—gas chromatography-mass spectrometry; HS-SPME—headspace-solid-phase microextraction; CAR/PDMS—Carboxen/Polydimethylsiloxane; PDMS/DVB—Polydimethylsiloxane/Divinylbenzene; GC-FID—gas chromatography with flame ionization detection; GC-IMS—gas chromatography coupled with ion mobility spectrometry; GC-TOFMS—gas chromatography/time-of-flight mass spectrometry; PCA—principal component analysis; OPLS-DA—orthogonal partial least squares discriminant analysis; ANOVA—analysis of variance; AMOVA—analysis of molecular variance; LSD—least significant difference; ROC—receiver operating characteristic; FDA—Fisher discriminant analysis; KNN—k-nearest neighbors algorithm; PLS-DA—partial least squares discriminant analysis; PNN—probabilistic neural network; LDA—linear discriminant analysis; DFA—discriminant function analysis; MSTFA—*N*-methyl-*N*-(trimethylsilyl)trifluoroacetamide; SCFA—short-chain fatty acid; PFBB—pentafluorobenzyl bromide; BSTFA—*N*,*O*-bis(trimethylsilyl)trifluoroacetamide; TMCS—trimethylsilyl chloride; ECF—ethyl chloroformate; EDIPA—3’-*O*-ethyl-*N*,*N*-diisopropylphosphoramidite; SNM—screening of nonvolatile metabolites; SVM—screening of volatile metabolites.

**Table 2:** List of the most relevant compounds reported as potential biomarkers of CRC.

Compound	CAS Number	Code	Matrix	Reference
**ALCOHOLS, POLYOLS AND PHENOLS**				
1-octanol	111-87-5	M1	Urine↓	Silva et al., 2011 [32]
hexen-1-ol	928-95-0	M2	Urine	Arasaradnam et al., 2014 [34]
2-propanol	67-63-0	M3	Feces↑	Bond et al., 2019 [43]
4-methylphenol (*p*-cresol)	106-44-5	M4	Urine↑ Urine↑ Urine↓_post_ Urine↓ Urine↑	Silva et al., 2011 [32] Qiu et al., 2010 [31] Qiu et al., 2010 [31] -Cheng et al., 2012 [33] Liesenfeld et al., 2015 [35]
ethanol	64-17-5	M5	Breath↓	Amal et al., 2016 [51]
glycerol (glycerin)	56-81-5	M6	Feces↓ Feces↓	Weir et al., 2013 [38] Wang et al., 2017 [41]
monoacyl glycerol	-	M7	Feces↓	Wang et al., 2017 [41]
phenol	108-95-2	M8	Urine↓	Cheng et al., 2012 [33]
guaiacol	90-05-1	M9	Urine↓	Liesenfeld et al., 2015 [35]
2,3-butanediol	513-85-9	M10	Urine↓	Liesenfeld et al., 2015 [35]
**ALDEHYDES**				
acetaldehyde	75-07-0	M11	Urine	Arasaradnam et al., 2014 [34]
decanal	112-31-2	M12	Breath↑ Breath↑	Altomare et al., 2013 [47] Altomare et al., 2015 [50]
heptanal	111-71-7	M13	Urine↓	Silva et al., 2011 [32]
hexanal	66-25-1	M14	Urine↓ Urine	Silva et al., 2011 [32] Arasaradnam et al., 2014 [34]
nonanal	124-19-6	M15	Breath↑ Breath↑	Altomare et al., 2013 [47] Altomare et al., 2015 [50]
**KETONES**				
2-hexanone	591-78-6	M16	Feces↑	Bond et al., 2019 [43]
3-heptanone	106-35-4	M17	Urine↑ Urine	Silva et al., 2011 [32] Arasaradnam et al., 2014 [34]
4-heptanone	123-19-3	M18	Urine	Arasaradnam et al., 2014 [34]
4-methyl-2-pentanone	108-10-1	M19	Breath↑ Breath↑	Altomare et al., 2013 [47] Altomare et al., 2015 [50]
2-pentanone	107-87-9	M20	Urine	Arasaradnam et al., 2014 [34]
acetone	67-64-1	M21	Breath↑ Urine	Amal et al., 2016 [51] Arasaradnam et al., 2014 [34]
2,3-butanedione	431-03-8	M22	Urine	Arasaradnam et al., 2014 [34]
**ESTERS**				
phenyl acetate	122-79-2	M23	Urine↑ Urine↓_post_	Qiu et al., 2010 [31] Qiu et al., 2010 [31]
**ETHERS**				
anisole	100-66-3	M24	Urine↑	Silva et al., 2011 [32]
**HYDROCARBONS**				
methane	74-82-8	M25	Breath Breath	Haines et al. 1977 [44] Piqué et al. 1984 [45]
1,2-pentadiene	591-95-7	M26	Breath↑ Breath↑	Altomare et al., 2013 [47] Altomare et al., 2015 [50]
1,3-dimethylbenzene	108-38-3	M27	Breath↑ Breath↓ Breath↑	Altomare et al., 2013 [47] Peng et al., 2010 [46] Altomare et al., 2015 [50]
1,4-dimethylbenzene (1,4-xylene)	106-42-3	M28	Breath↑ Breath↑ Feces↑	Altomare et al., 2013 [47] Altomare et al., 2015 [50] Bond et al., 2019 [43]
1-octene	111-66-0	M29	Breath↑	Altomare et al., 2015 [50]
2-methylbutane	78-78-4	M30	Breath↑ Breath↑	Altomare et al., 2013 [47] Altomare et al., 2015 [50]
2-methylpentane	107-83-5	M31	Breath↑ Breath↑	Altomare et al., 2013 [47] Altomare et al., 2015 [50]
3-methylpentane	96-14-0	M32	Breath↑	Altomare et al., 2013 [47]
octane	111-65-9	M33	Breath↑	Altomare et al., 2015 [50]
undecane	1120-21-4	M34	Breath↑	Altomare et al., 2015 [50]
4-methyloctane	2216-34-4	M35	Breath↑ Breath↓	Altomare et al., 2013 [47] Amal et al., 2016 [51]
cyclohexane	110-82-7	M36	Breath↑ Breath↑	Altomare et al., 2013 [47] Altomare et al., 2015 [50]
dodecane	112-40-3	M37	Breath↑ Breath↑	Wang et al., 2014 [49] Altomare et al., 2015 [50]
heptane	142-82-5	M38	Breath↑	Altomare et al., 2015 [50]
methylcyclohexane	108-87-2	M39	Breath↑ Breath↑	Altomare et al., 2013 [47] Altomare et al., 2015 [50]
methylcyclopentane	96-37-7	M40	Breath↑ Breath↑	Altomare et al., 2013 [47] Altomare et al., 2015 [50]
p-cymene	99-87-6	M41	Urine↑	Silva et al., 2011 [32]
*γ*-terpinene	99-85-4	M42	Urine↑	Silva et al., 2011 [32]
beta-pinene	127-91-3	M43	Breath↑	Altomare et al., 2015 [50]
**ACIDS**				
acetic acid	64-19-7	M44	Feces↑ Feces↑	Weir et al., 2013 [38] Wang et al., 2017 [41]
benzeneacetic acid (phenylacetic acid)	103-82-2	M45	Feces↑ Feces↑	Weir et al., 2013 [38] Wang et al., 2017 [41]
butyric acid	107-92-6	M46	Feces↓ Feces↑	Weir et al., 2013 [38] Wang et al., 2017 [41]
citric acid	77-92-9	M47	Urine↓ Urine↓_post_ Urine↓ Urine↑	Qiu et al., 2010 [31] Qiu et al., 2010 [31] Cheng et al., 2012 [33] Liesenfeld et al., 2015 [35]
elaidic acid	112-79-8	M48	Feces↓ Feces↓	Weir et al., 2013 [38] Wang et al., 2017 [41]
isobutyric acid	79-31-2	M49	Feces↑ Feces↓	Weir et al., 2013 [38] Wang et al., 2017 [41]
2-hydroxyisobutyric acid	594-61-6	M50	Urine↑	Liesenfeld et al., 2015 [35]
isocitric acid	320-77-4	M51	Urine↓	Qiu et al., 2010 [31]
isovaleric acid	503-74-2	M52	Feces↑ Feces↑	Weir et al., 2013 [38] Wang et al., 2017 [41]
3-hydroxybutanoic acid	300-85-6	M53	Urine↑	Liesenfeld et al., 2015 [35]
lactic acid	50-21-5	M54	Urine↑	Liesenfeld et al., 2015 [35]
linoleic acid	60-33-3	M55	Feces↓ Feces↓ Feces↑^m^ Feces↓	Weir et al., 2013 [38] Wang et al., 2017 [41] Song et al., 2018 [42] Phua et al., 2014 [39]
myristic acid	544-63-8	M56	Feces↑ Feces↓ Urine↓	Weir et al., 2013 [38] Wang et al., 2017 [41] Cheng et al., 2012 [33]
oleic acid	2027-47-6	M57	Feces↓ Feces↓ Feces↑^m^	Weir et al., 2013 [38] Wang et al., 2017 [41] Song et al., 2018 [42]
oxalic acid	6153-56-6	M58	Urine Urine↓	Arasaradnam et al., 2014 [34] Liesenfeld et al., 2015 [35]
propionic acid	79-09-4	M59	Feces↑ Feces↑	Weir et al., 2013 [38] Wang et al., 2017 [41]
pyruvic acid	127-17-3	M60	Urine↓	Cheng et al., 2012 [33]
succinic acid	110-15-6	M61	Urine↓	Qiu et al., 2010 [31]
valeric acid	109-52-4	M62	Feces↑ Feces↑	Weir et al., 2013 [38] Wang et al., 2017 [41]
fumaric acid	110-17-8	M63	Urine↑	Cheng et al., 2012 [33]
**SULFUR-CONTAINING COMPOUNDS**				
2-methoxythiophene	16839-97-7	M64	Urine↑	Silva et al., 2011 [32]
dimethyl disulfide	624-92-0	M65	Urine↓	Silva et al., 2011 [32]
**NITROGEN-CONTAINING COMPOUNDS**				
putrescine	110-60-1	M66	Urine↑	Cheng et al., 2012 [33]
dimethyl-thiourea	534-13-4	M67	Urine	Arasaradnam et al., 2014 [34]
allyl isothiocyanate	57-06-7	M68	Urine	Arasaradnam et al., 2014 [34]
**AMINO ACIDS AND THEIR DERIVATIVES**				
2-aminobutyric acid	1492-24-6	M69	Urine↑	Cheng et al., 2012 [33]
hippuric acid	495-69-2	M70	Urine↓_post_ Urine↓	Qiu et al., 2010 [31] Cheng et al., 2012 [33]
5-oxoproline	149-87-1	M71	Urine↑ Urine↑_post_	Qiu et al., 2010 [31] Qiu et al., 2010 [31]
alanine	56-41-7	M72	Feces↑ Urine↓ Urine↓	Weir et al., 2013 [38] Cheng et al., 2012 [33] Liesenfeld et al., 2015 [35]
aspartic acid	56-84-8	M73	Feces↑ Feces↑	Weir et al., 2013 [38] Wang et al., 2017 [41]
glutamic acid	617-65-2	M74	Feces↑ Feces↑ Urine↑ Urine↑	Weir et al., 2013 [38] Wang et al., 2017 [41] Qiu et al., 2010 [31] Liesenfeld et al., 2015 [35]
glutamine	56-85-9	M75	Urine↓	Liesenfeld et al., 2015 [35]
glycine	56-40-6	M76	Feces↑ Feces↑	Weir et al., 2013 [38] Wang et al., 2017 [41]
histidine	71-00-1	M77	Urine↓ Urine↑_post_ Urine↓	Qiu et al., 2010 [31] Qiu et al., 2010 [31] Liesenfeld et al., 2015 [35]
isoleucine	73-32-5	M78	Urine↑_post_	Qiu et al., 2010 [31]
leucine	61-90-5	M79	Feces↑ Feces↑ Urine↑_post_	Weir et al., 2013 [38] Wang et al., 2017 [41] Qiu et al., 2010 [31]
lysine	70-54-2	M80	Feces↑ Urine↑_post_ Urine↓	Weir et al., 2013 [38] Qiu et al., 2010 [31] Liesenfeld et al., 2015 [35]
phenylacetylglutamine	28047-15-6	M81	Urine↑ Urine↓_post_	Qiu et al., 2010 [31] Qiu et al., 2010 [31]
phenylalanine	150-30-1	M82	Feces↑ Feces↑ Urine↓	Weir et al., 2013 [38] Wang et al., 2017 [41] Liesenfeld et al., 2015 [35]
proline	609-36-9	M83	Feces↑ Feces↑	Weir et al., 2013 [38] Wang et al., 2017 [41]
salicyluric acid (2-hydroxyhippuric acid)	487-54-7	M84	Urine↑ Urine↓_post_ Urine↓	Qiu et al., 2010 [31] Qiu et al., 2010 [31] Liesenfeld et al., 2015 [35]
serine	56-45-1	M85	Feces↑ Feces↑ Urine↑_post_	Weir et al., 2013 [38] Wang et al., 2017 [41] Qiu et al., 2010 [31]
threonine	72-19-5	M86	Feces↑ Urine↑_post_ Urine↓	Weir et al., 2013 [38] Qiu et al., 2010 [31] Liesenfeld et al., 2015 [35]
tyrosine	60-18-4	M87	Urine↑_post_ Urine↓	Qiu et al., 2010 [31] Liesenfeld et al., 2015 [35]
tryptophan	54-12-6	M88	Urine↑ Urine↑_post_	Qiu et al., 2010 [31] Qiu et al., 2010 [31]
valine	516-06-3	M89	Feces↑ Feces↑	Weir et al., 2013 [38] Wang et al., 2017 [41]
**SUGARS AND THEIR DERIVATIVES**				
fructose	7660-25-5	M90	Feces↓ Urine↓	Phua et al., 2014 [39] Liesenfeld et al., 2015 [35]
xylose	58-86-6	M91	Urine↓	Cheng et al., 2012 [33]
sorbose	87-79-6	M92	Urine↓	Cheng et al., 2012 [33]
arabitol	7643-75-6	M93	Urine↓	Cheng et al., 2012 [33]
arabinose	147-81-9	M94	Urine↓	Liesenfeld et al., 2015 [35]
mannitol	69-65-8	M95	Urine↓	Liesenfeld et al., 2015 [35]
glucuronic acid	6556-12-3	M96	Urine↓	Cheng et al., 2012 [33]
gluconic acid	526-95-4	M97	Urine↓	Liesenfeld et al., 2015 [35]
threonic acid	3909-12-4	M98	Urine↓	Cheng et al., 2012 [33]
3-phosphoglyceric acid	820-11-1	M99	Urine↓	Liesenfeld et al., 2015 [35]
**COMPLEX NITROGEN COMPOUNDS AND THEIR DERIVATIVES**				
uracil	66-22-8	M100	Urine↓	Cheng et al., 2012 [33]
xanthine	69-89-6	M101	Urine↑	Liesenfeld et al., 2015 [35]
**STEROIDS AND THEIR DERIVATIVES**				
cholesterol derivative	-	M102	Feces↑ Feces↑	Weir et al., 2013 [38] Wang et al., 2017 [41]
ursodeoxycholic acid	128-13-2	M103	Feces↓ Feces↓	Weir et al., 2013 [38] Wang et al., 2017 [41]
**OTHERS**				
pantothenic acid (vitamin B_5_)	599-54-2	M104	Feces↑ Feces↓	Weir et al., 2013 [38] Wang et al., 2017 [41]

where: ↑—concentration elevated in comparison of healthy controls; ↓—concentration decreased in comparison of healthy controls; _post_—index regarding postoperative samples; ^m^—index regarding only male samples; no arrows—changes in concentration of compound not mentioned by authors. Code—reference used for further discussions in Section 3.

**Table 3:** Incidence distribution of potential CRC biomarkers in all three matrices, according to functional group.

Incidence of Compounds, Per Class	Breath	Fecal Samples	Urine	All
Totals	65	64	148	278
Alcohols, polyols and phenols	4	6	12	22
Aldehydes	4	0	5	9
Ketones	4	1	8	13
Esters	3	4	6	13
Ethers	0	0	3	3
Hydrocarbons	47	1	8	56
Acids	1	25	27	53
Sulfur-containing compounds	0	0	2	2
Nitrogen-containing compounds	2	0	11	13
Amino acids and their derivatives	0	19	47	66
Sugars and their derivatives	0	1	15	16
Complex nitrogen compounds and derivatives	0	0	4	5
Steroids and their derivatives	0	4	0	4
Others	0	3	0	3

## Supplementary Materials

The following are available online at https://www.mdpi.com/2077-0383/9/10/3191/s1. Table S1: Total number of compounds reported as potential biomarkers of CRC.
