# Supplementary material for: A Review of GC-Based Analysis of Non-Invasive Biomarkers of Colorectal Cancer and Related Pathways
Source: J Clin Med. 2020 Oct 1;9(10):3191. doi: 10.3390/jcm9103191 (PMC7601558; doi:10.3390/jcm9103191)
Supplement: Supplementary file 1 [file jcm-09-03191-s001.pdf]

Table S1. Total number of compounds reported as potential biomarkers of CRC, where: ↑ – concentration elevated in comparison of healthy controls; ↓ – concentration decreased in comparison of healthy controls; $_{post}$ – index regarding postoperative samples; ^{m} – index regarding only male samples; no arrows – changes of concentration of compound not mentioned by authors; GC-MS – gas chromatography-mass spectrometry; dHS-SPME-GC-qMS – dynamic headspace solid-phase microextraction gas chromatography-quadrupole mass spectrometry; HS-SPME-GC-MS – headspace solid-phase microextraction gas chromatography-mass spectrometry; TD-GC-MS – thermal desorption gas chromatography-mass spectrometry; GC-FID – gas chromatography-flame ionization detector; GC-TOFMS – gas chromatography-time-of-flight mass spectrometry; U – urine; F – feces; Br – breath.

Compound	Matrix			Method	Reference
	Breath	Feces	Urine		
**ALCOHOLS, POLYOLS AND PHENOLS**					
1-octanol			U↓	dHS-SPME-GC-qMS	[Silva et al 2011]
hexen-1-ol			U	in-tube extraction-GC-MS	[Arasaradnam et al 2014]
2-propanol		F↑		HS-SPME-GC-MS	[Bond et al 2019]
4-ethyl-1-octyn-3-ol	Br↑			HS-SPME-GC-MS	[Wang et al 2014]
4-methylphenol (*p*-cresol)			U↑	dHS-SPME-GC-qMS	[Silva et al 2011]
			U↑	solvent extraction GC-MS	[Qiu et al 2010]
			U↓$_{post}$	solvent extraction GC-MS	[Qiu et al 2010]
			U↓	solvent extraction GC-TOFMS	[Cheng et al 2012]
			U↑	solvent extraction GC-MS	[Liesenfeld et al 2015]
5-methyl-2-propan-2-yl-cyclohexan-1-ol		F↓		HS-SPME-GC-MS	[Bond et al 2019]
cyclooctylmethanol	Br↑			HS-SPME-GC-MS	[Wang et al 2014]
ethanol	Br↓			TD-GC-MS	[Amal et al 2016]
glycerol (glycerin)		F↓		solvent extraction GC-MS	[Weir et al 2013]
		F↓		solvent extraction GC-MS	[Wang et al 2017]
monoacyl glycerol		F↓		solvent extraction GC-MS	[Wang et al 2017]
monooleoylglycerol		F↓		solvent extraction GC-MS	[Weir et al 2013]
phenol			U↓	solvent extraction GC-TOFMS	[Cheng et al 2012]
guaiacol			U↓	solvent extraction GC-MS	[Liesenfeld et al 2015]
hydroquinone (benzene-1,4-diol)			U↓	solvent extraction GC-MS	[Liesenfeld et al 2015]
trans-2-dodecen-1-ol	Br↑			HS-SPME-GC-MS	[Wang et al 2014]
2,3-butanediol			U↓	solvent extraction GC-MS	[Liesenfeld et al 2015]
pyrogallol			U↓	solvent extraction GC-MS	[Liesenfeld et al 2015]
**ALDEHYDES**					
acetaldehyde			U	in-tube extraction-GC-MS	[Arasaradnam et al 2014]
2-methyl-3-phenyl-2-propenal			U↑	dHS-SPME-GC-qMS	[Silva et al 2011]
decanal	Br↑			TD-GC-MS	[Altomare et al 2013]

	Br↑			TD-GC-MS	[Altomare et al 2015]
heptanal			U↓	dHS-SPME-GC-qMS	[Silva et al 2011]
hexanal			U↓	dHS-SPME-GC-qMS	[Silva et al 2011]
			U	in-tube extraction-GC-MS	[Arasaradnam et al 2014]
nonanal	Br↑			TD-GC-MS	[Altomare et al 2013]
	Br↑			TD-GC-MS	[Altomare et al 2015]
**KETONES**					
2-hexanone		F↑		HS-SPME-GC-MS	[Bond et al 2019]
3-heptanone			U↑	dHS-SPME-GC-qMS	[Silva et al 2011]
			U	in-tube extraction-GC-MS	[Arasaradnam et al 2014]
4-heptanone			U	in-tube extraction-GC-MS	[Arasaradnam et al 2014]
2,4-dimethyl-3-pentanone			U	in-tube extraction-GC-MS	[Arasaradnam et al 2014]
4-methyl-2-pentanone	Br↑			TD-GC-MS	[Altomare et al 2013]
	Br↑			TD-GC-MS	[Altomare et al 2015]
2-pentanone			U	in-tube extraction-GC-MS	[Arasaradnam et al 2014]
acetone	Br↑			TD-GC-MS	[Amal et al 2016]
			U	in-tube extraction-GC-MS	[Arasaradnam et al 2014]
3-methyl-2-butanone			U	in-tube extraction-GC-MS	[Arasaradnam et al 2014]
cyclohexanone	Br↑			HS-SPME-GC-MS	[Wang et al 2014]
2,3-butanedione			U	in-tube extraction-GC-MS	[Arasaradnam et al 2014]
**ESTERS**					
3-hydroxy-2,4,4-trimethylpentyl 2-methylpropanoate	Br↑			HS-SPME-GC-MS	[Wang et al 2014]
4-(4-propylcyclohexyl)-4′-cyano[1,1′-biphenyl]-4-yl ester benzoic acid	Br↓			HS-SPME-GC-MS	[Peng et al 2010]
ethyl 3-methylbutanoate		F↑		HS-SPME-GC-MS	[Bond et al 2019]
ethyl acetate	Br↑			TD-GC-MS	[Amal et al 2016]
phenyl acetate			U↑	solvent extraction GC-MS	[Qiu et al 2010]
			U$\downarrow_{post}$	solvent extraction GC-MS	[Qiu et al 2010]
propan-2-yl butanoate		F↑		HS-SPME-GC-MS	[Bond et al 2019]
propan-2-yl pentanoate		F↑		HS-SPME-GC-MS	[Bond et al 2019]
propan-2-yl propanoate		F↑		HS-SPME-GC-MS	[Bond et al 2019]
pentyl ethylbenzoate (ethylbenzoic acid, pentyl ester)			U	in-tube extraction-GC-MS	[Arasaradnam et al 2014]
methyl carbamate (carbamic acid, methyl ester)			U	in-tube extraction-GC-MS	[Arasaradnam et al 2014]
3-indoxylsulfate			U↑	solvent extraction GC-MS	[Liesenfeld et al 2015]
5-hydroxyindole acetate			U↓	solvent extraction GC-MS	[Liesenfeld et al 2015]
**ETHERS**					
ethylene oxide			U	in-tube extraction-GC-MS	[Arasaradnam et al 2014]
anisole			U↑	dHS-SPME-GC-qMS	[Silva et al 2011]
oxepane			U	in-tube extraction-GC-MS	[Arasaradnam et al 2014]
**HYDROCARBONS**					
methane	Br			direct gas sampling GC	[Haines et al 1977]
	Br			direct gas sampling GC-FID	[Piqué et al 1984]

1,1′-(1-butenylidene)bis benzene	Br↑			HS-SPME-GC-MS	[Peng et al 2010]
1,2,3-trimethylbenzene	Br↑			TD-GC-MS	[Altomare et al 2015]
1,2,4-trimethylbenzene			U↑	dHS-SPME-GC-qMS	[Silva et al 2011]
1,2-dihydro-1,1,6-trimethylnaphthalene			U↑	dHS-SPME-GC-qMS	[Silva et al 2011]
1,2-pentadiene	Br↑			TD-GC-MS	[Altomare et al 2013]
	Br↑			TD-GC-MS	[Altomare et al 2015]
5-butylnonane	Br↑			TD-GC-MS	[Altomare et al 2015]
propylbenzene	Br↑			TD-GC-MS	[Altomare et al 2015]
1,3-dimethylbenzene	Br↑			TD-GC-MS	[Altomare et al 2013]
	Br↓			HS-SPME-GC-MS	[Peng et al 2010]
	Br↑			TD-GC-MS	[Altomare et al 2015]
1,4,5-trimethylnaphthalene			U↑	dHS-SPME-GC-qMS	[Silva et al 2011]
1-methylnaphthalene	Br↑			TD-GC-MS	[Altomare et al 2015]
2-methylnaphthalene	Br↑			TD-GC-MS	[Altomare et al 2015]
1,4-dimethylbenzene (1,4-xylene)	Br↑			TD-GC-MS	[Altomare et al 2013]
	Br↑			TD-GC-MS	[Altomare et al 2015]
		F↑		HS-SPME-GC-MS	[Bond et al 2019]
1-iodo nonane	Br↑			HS-SPME-GC-MS	[Peng et al 2010]
1-octene	Br↑			TD-GC-MS	[Altomare et al 2015]
4-methyl-1-hexene			U	in-tube extraction-GC-MS	[Arasaradnam et al 2014]
2,2-dimethyldecane	Br↑			HS-SPME-GC-MS	[Wang et al 2014]
2-methylbutane	Br↑			TD-GC-MS	[Altomare et al 2013]
	Br↑			TD-GC-MS	[Altomare et al 2015]
2-methylpentane	Br↑			TD-GC-MS	[Altomare et al 2013]
	Br↑			TD-GC-MS	[Altomare et al 2015]
3-methylpentane	Br↑			TD-GC-MS	[Altomare et al 2013]
octane	Br↑			TD-GC-MS	[Altomare et al 2015]
undecane	Br↑			TD-GC-MS	[Altomare et al 2015]
4-methyloctane	Br↑			TD-GC-MS	[Altomare et al 2013]
	Br↓			TD-GC-MS	[Amal et al 2016]
4-methylundecane	Br↑			TD-GC-MS	[Altomare et al 2013]
6-*t*-butyl-2,2,9,9-tetramethyl-3,5-decadien-7-yne	Br↓			HS-SPME-GC-MS	[Wang et al 2014]
1,3-dimethyl-5-(1-methylethyl)benzene	Br↑			TD-GC-MS	[Altomare et al 2015]
1-methyl-3-(1-methylethyl)benzene	Br↑			TD-GC-MS	[Altomare et al 2015]
1-(1-methylethenyl)-2-(1-methylethyl)benzene	Br↑			TD-GC-MS	[Altomare et al 2015]
1-ethyl-1,2,4-trimethylbenzene	Br↑			TD-GC-MS	[Altomare et al 2015]
1-ethyl-2,4,5-trimethylbenzene	Br↑			TD-GC-MS	[Altomare et al 2015]
2,3-dihydro-1,6-dimethyl-1H-indene	Br↑			TD-GC-MS	[Altomare et al 2015]
2,3-dihydro-4,7-dimethyl-1H-indene	Br↑			TD-GC-MS	[Altomare et al 2015]
bornylene			U↑	dHS-SPME-GC-qMS	[Silva et al 2011]
cyclohexane	Br↑			TD-GC-MS	[Altomare et al 2013]

	Br↑			TD-GC-MS	[Altomare et al 2015]
dodecane	Br↑			HS-SPME-GC-MS	[Wang et al 2014]
	Br↑			TD-GC-MS	[Altomare et al 2015]
heptane	Br↑			TD-GC-MS	[Altomare et al 2015]
styrene			U	in-tube extraction-GC-MS	[Arasaradnam et al 2014]
methylcyclohexane	Br↑			TD-GC-MS	[Altomare et al 2013]
	Br↑			TD-GC-MS	[Altomare et al 2015]
methylcyclopentane	Br↑			TD-GC-MS	[Altomare et al 2013]
	Br↑			TD-GC-MS	[Altomare et al 2015]
p-cymene			U↑	dHS-SPME-GC-qMS	[Silva et al 2011]
trimethyldecane	Br↑			TD-GC-MS	[Altomare et al 2013]
γ-terpinene			U↑	dHS-SPME-GC-qMS	[Silva et al 2011]
beta-pinene	Br↑			TD-GC-MS	[Altomare et al 2015]
**ACIDS**					
[(1,1-dimethylethyl)thio] acetic acid	Br↓			HS-SPME-GC-MS	[Peng et al 2010]
3-hydroxybutanoic acid (β-hydroxybutyric acid)			U↑	solvent extraction GC-MS	[Liesenfeld et al 2015]
4-hydroxybutanoic acid (γ-hydroxybutyric acid)			U↑	solvent extraction GC-TOFMS	[Cheng et al 2012]
acetic acid		F↑		solvent extraction GC-MS	[Weir et al 2013]
		F↑		solvent extraction GC-MS	[Wang et al 2017]
hydroxyacetic acid (glycolic acid)			U↓	solvent extraction GC-TOFMS	[Cheng et al 2012]
benzeneacetic acid (phenylacetic acid)		F↑		solvent extraction GC-MS	[Weir et al 2013]
		F↑		solvent extraction GC-MS	[Wang et al 2017]
butyric acid		F↓		solvent extraction GC-MS	[Weir et al 2013]
		F↑		solvent extraction GC-MS	[Wang et al 2017]
citric acid			U↓	solvent extraction GC-MS	[Qiu et al 2010]
			U$\downarrow_{post}$	solvent extraction GC-MS	[Qiu et al 2010]
			U↓	solvent extraction GC-TOFMS	[Cheng et al 2012]
			U↑	solvent extraction GC-MS	[Liesenfeld et al 2015]
aconitic acid			U↓	solvent extraction GC-MS	[Qiu et al 2010]
elaidic acid		F↓		solvent extraction GC-MS	[Weir et al 2013]
		F↓		solvent extraction GC-MS	[Wang et al 2017]
isobutyric acid		F↑		solvent extraction GC-MS	[Weir et al 2013]
		F↓		solvent extraction GC-MS	[Wang et al 2017]
2-hydroxyisobutyric acid			U↑	solvent extraction GC-MS	[Liesenfeld et al 2015]
isocitric acid			U↓	solvent extraction GC-MS	[Qiu et al 2010]
isovaleric acid		F↑		solvent extraction GC-MS	[Weir et al 2013]
		F↑		solvent extraction GC-MS	[Wang et al 2017]
3-deoxypentanoic acid			U↓	solvent extraction GC-MS	[Liesenfeld et al 2015]
2,3-dihydroxybutyric acid			U↑	solvent extraction GC-MS	[Liesenfeld et al 2015]
2-deoxytetronic acid (3,4-dihydroxybutyric acid)			U↓	solvent extraction GC-MS	[Liesenfeld et al 2015]

lactic acid			U↑	solvent extraction GC-MS	[Liesenfeld et al 2015]
linoleic acid		F↓		solvent extraction GC-MS	[Weir et al 2013]
		F↓		solvent extraction GC-MS	[Wang et al 2017]
		F↑[m]		solvent extraction GC-MS	[Song et al 2018]
		F↓		solvent extraction GC-TOFMS	[Phua et al 2014]
myristic acid		F↑		solvent extraction GC-MS	[Weir et al 2013]
		F↓		solvent extraction GC-MS	[Wang et al 2017]
			U↓	solvent extraction GC-TOFMS	[Cheng et al 2012]
oleic acid		F↓		solvent extraction GC-MS	[Weir et al 2013]
		F↓		solvent extraction GC-MS	[Wang et al 2017]
		F↑[m]		solvent extraction GC-MS	[Song et al 2018]
oxalic acid			U	in-tube extraction-GC-MS	[Arasaradnam et al 2014]
			U↓	solvent extraction GC-MS	[Liesenfeld et al 2015]
p-hydroxyphenylacetic acid			U↑	solvent extraction GC-MS	[Qiu et al 2010]
propionic acid		F↑		solvent extraction GC-MS	[Weir et al 2013]
		F↑		solvent extraction GC-MS	[Wang et al 2017]
pyruvic acid			U↓	solvent extraction GC-TOFMS	[Cheng et al 2012]
succinic acid			U↓	solvent extraction GC-MS	[Qiu et al 2010]
valeric acid		F↑		solvent extraction GC-MS	[Weir et al 2013]
		F↑		solvent extraction GC-MS	[Wang et al 2017]
fumaric acid			U↑	solvent extraction GC-TOFMS	[Cheng et al 2012]
2-aminomalonic acid			U↑	solvent extraction GC-MS	[Qiu et al 2010]
pentanedioic acid (glutaric acid)			U↓	solvent extraction GC-MS	[Qiu et al 2010]
2-hydroxyglutaric acid			U↑	solvent extraction GC-MS	[Liesenfeld et al 2015]
maleamic acid			U↓	solvent extraction GC-MS	[Liesenfeld et al 2015]
tartaric acid			U↓	solvent extraction GC-MS	[Liesenfeld et al 2015]
cis-4-hydroxycyclohexylcarboxylic acid			U↓	solvent extraction GC-MS	[Liesenfeld et al 2015]
**SULFUR-CONTAINING COMPOUNDS**					
2-methoxythiophene			U↑	dHS-SPME-GC-qMS	[Silva et al 2011]
dimethyl disulfide			U↓	dHS-SPME-GC-qMS	[Silva et al 2011]
**NITROGEN-CONTAINING COMPOUNDS**					
2,7-dimethylquinoline			U↑	dHS-SPME-GC-qMS	[Silva et al 2011]
2-amino-5-isopropyl-8-methyl-1-azulenecarbonitrile	Br↓			HS-SPME-GC-MS	[Peng et al 2010]
ethylaniline	Br↑			HS-SPME-GC-MS	[Wang et al 2014]
putrescine			U↑	solvent extraction GC-TOFMS	[Cheng et al 2012]
5-methylhydantoine			U↓	solvent extraction GC-MS	[Liesenfeld et al 2015]
dimethyl-thiourea			U	in-tube extraction-GC-MS	[Arasaradnam et al 2014]
allyl isothiocyanate			U	in-tube extraction-GC-MS	[Arasaradnam et al 2014]
methoxy-phenyl-oxime			U	in-tube extraction-GC-MS	[Arasaradnam et al 2014]
isothiocyanato-cyclopropane			U	in-tube extraction-GC-MS	[Arasaradnam et al 2014]
2-cyano-acetamide			U	in-tube extraction-GC-MS	[Arasaradnam et al 2014]

dimethyl diazene	U	in-tube extraction-GC-MS	[Arasaradnam et al 2014]
cyclobutylamine	U	in-tube extraction-GC-MS	[Arasaradnam et al 2014]
dopamine	U↓	solvent extraction GC-MS	[Liesenfeld et al 2015]
**AMINO ACIDS AND THEIR DERIVATIVES**			
asparagine	U↑	solvent extraction GC-MS	[Qiu et al 2010]
2-ethylhydracrylic acid	U↑	solvent extraction GC-MS	[Delphan et al 2018]
2-methyl-3-hydroxybutyric acid	U↑	solvent extraction GC-MS	[Delphan et al 2018]
2-aminobutyric acid	U↑	solvent extraction GC-TOFMS	[Cheng et al 2012]
3-methylhistidine	U↓	solvent extraction GC-MS	[Qiu et al 2010]
1-methylhistidine	U↓	solvent extraction GC-MS	[Liesenfeld et al 2015]
hippuric acid	U↓$_{post}$	solvent extraction GC-MS	[Qiu et al 2010]
	U↓	solvent extraction GC-TOFMS	[Cheng et al 2012]
5-hydroxyindoleacetic acid	U↑	solvent extraction GC-MS	[Qiu et al 2010]
indole-3-acetic acid	U↓	solvent extraction GC-MS	[Liesenfeld et al 2015]
5-hydroxytryptophan	U↑	solvent extraction GC-MS	[Qiu et al 2010]
5-oxoproline	U↑	solvent extraction GC-MS	[Qiu et al 2010]
	U↑$_{post}$	solvent extraction GC-MS	[Qiu et al 2010]
pyrrole-2-carboxylic acid	U↓	solvent extraction GC-MS	[Liesenfeld et al 2015]
alanine	F↑	solvent extraction GC-MS	[Weir et al 2013]
	U↓	solvent extraction GC-TOFMS	[Cheng et al 2012]
	U↓	solvent extraction GC-MS	[Liesenfeld et al 2015]
aspartic acid	F↑	solvent extraction GC-MS	[Weir et al 2013]
	F↑	solvent extraction GC-MS	[Wang et al 2017]
glutamic acid	F↑	solvent extraction GC-MS	[Weir et al 2013]
	F↑	solvent extraction GC-MS	[Wang et al 2017]
	U↑	solvent extraction GC-MS	[Qiu et al 2010]
	U↑	solvent extraction GC-MS	[Liesenfeld et al 2015]
glutamine	U↓	solvent extraction GC-MS	[Liesenfeld et al 2015]
homovanillic acid	U↓	solvent extraction GC-TOFMS	[Cheng et al 2012]
glycine	F↑	solvent extraction GC-MS	[Weir et al 2013]
	F↑	solvent extraction GC-MS	[Wang et al 2017]
histidine	U↓	solvent extraction GC-MS	[Qiu et al 2010]
	U↑$_{post}$	solvent extraction GC-MS	[Qiu et al 2010]
	U↓	solvent extraction GC-MS	[Liesenfeld et al 2015]
isoleucine	U↑$_{post}$	solvent extraction GC-MS	[Qiu et al 2010]
leucine	F↑	solvent extraction GC-MS	[Weir et al 2013]
	F↑	solvent extraction GC-MS	[Wang et al 2017]
	U↑$_{post}$	solvent extraction GC-MS	[Qiu et al 2010]
lysine	F↑	solvent extraction GC-MS	[Weir et al 2013]
	U↑$_{post}$	solvent extraction GC-MS	[Qiu et al 2010]
	U↓	solvent extraction GC-MS	[Liesenfeld et al 2015]

N-acetylaspartic acid		U↑	solvent extraction GC-MS	[Qiu et al 2010]
phenylacetylglutamine		U↑	solvent extraction GC-MS	[Qiu et al 2010]
		$U\downarrow_{post}$	solvent extraction GC-MS	[Qiu et al 2010]
phenylalanine	F↑		solvent extraction GC-MS	[Weir et al 2013]
	F↑		solvent extraction GC-MS	[Wang et al 2017]
		U↓	solvent extraction GC-MS	[Liesenfeld et al 2015]
proline	F↑		solvent extraction GC-MS	[Weir et al 2013]
	F↑		solvent extraction GC-MS	[Wang et al 2017]
glycylproline		U↑	solvent extraction GC-MS	[Qiu et al 2010]
salicyluric acid (2-hydroxyhippuric acid)		U↑	solvent extraction GC-MS	[Qiu et al 2010]
		$U\downarrow_{post}$	solvent extraction GC-MS	[Qiu et al 2010]
		U↓	solvent extraction GC-MS	[Liesenfeld et al 2015]
3-hydroxyhippuric acid		U↓	solvent extraction GC-MS	[Liesenfeld et al 2015]
4-hydroxyhippuric acid		U↓	solvent extraction GC-MS	[Liesenfeld et al 2015]
methyl-*o*-hydroxyhippuric acid		U↓	solvent extraction GC-MS	[Qiu et al 2010]
serine	F↑		solvent extraction GC-MS	[Weir et al 2013]
	F↑		solvent extraction GC-MS	[Wang et al 2017]
		$U\uparrow_{post}$	solvent extraction GC-MS	[Qiu et al 2010]
threonine	F↑		solvent extraction GC-MS	[Weir et al 2013]
		$U\uparrow_{post}$	solvent extraction GC-MS	[Qiu et al 2010]
		U↓	solvent extraction GC-MS	[Liesenfeld et al 2015]
tyrosine		$U\uparrow_{post}$	solvent extraction GC-MS	[Qiu et al 2010]
		U↓	solvent extraction GC-MS	[Liesenfeld et al 2015]
tryptophan		U↑	solvent extraction GC-MS	[Qiu et al 2010]
		$U\uparrow_{post}$	solvent extraction GC-MS	[Qiu et al 2010]
xanthurenic acid		U↓	solvent extraction GC-MS	[Liesenfeld et al 2015]
valine	F↑		solvent extraction GC-MS	[Weir et al 2013]
	F↑		solvent extraction GC-MS	[Wang et al 2017]
γ-glutamyl valine		U↑	solvent extraction GC-MS	[Liesenfeld et al 2015]
**SUGARS AND THEIR DERIVATIVES**				
fructose	F↓		solvent extraction GC-TOFMS	[Phua et al 2014]
		U↓	solvent extraction GC-MS	[Liesenfeld et al 2015]
glyceryl glycoside		U↓	solvent extraction GC-MS	[Liesenfeld et al 2015]
myo-inositol		U↓	solvent extraction GC-MS	[Liesenfeld et al 2015]
xylose		U↓	solvent extraction GC-TOFMS	[Cheng et al 2012]
sorbose		U↓	solvent extraction GC-TOFMS	[Cheng et al 2012]
arabitol		U↓	solvent extraction GC-TOFMS	[Cheng et al 2012]
arabinose		U↓	solvent extraction GC-MS	[Liesenfeld et al 2015]
mannitol		U↓	solvent extraction GC-MS	[Liesenfeld et al 2015]
glucuronic acid		U↓	solvent extraction GC-TOFMS	[Cheng et al 2012]
gluconic acid		U↓	solvent extraction GC-MS	[Liesenfeld et al 2015]

threonic acid	U↓	solvent extraction GC-TOFMS	[Cheng et al 2012]
3-phosphoglyceric acid	U↓	solvent extraction GC-MS	[Liesenfeld et al 2015]
p-cresol-*β*-O-glucuronide	U↑	solvent extraction GC-MS	[Liesenfeld et al 2015]
2-O-glycerol-*α*-D-galactopyranoside	U↓	solvent extraction GC-MS	[Liesenfeld et al 2015]
galacturonic acid	U↓	solvent extraction GC-MS	[Liesenfeld et al 2015]
**COMPLEX NITROGEN COMPOUNDS AND THEIR DERIVATIVES**			
uracil	U↓	solvent extraction GC-TOFMS	[Cheng et al 2012]
xanthine	U↑	solvent extraction GC-MS	[Liesenfeld et al 2015]
pseudouridine	U↑	solvent extraction GC-MS	[Liesenfeld et al 2015]
acetyloxime-pyridine carboxaldehyde	U	in-tube extraction-GC-MS	[Arasaradnam et al 2014]
hydrocinnamoyl-bezene-ethanamine	U	in-tube extraction-GC-MS	[Arasaradnam et al 2014]
**STEROIDS AND THEIR DERIVATIVES**			
cholesterol derivative	F↑	solvent extraction GC-MS	[Weir et al 2013]
	F↑	solvent extraction GC-MS	[Wang et al 2017]
ursodeoxycholic acid	F↓	solvent extraction GC-MS	[Weir et al 2013]
	F↓	solvent extraction GC-MS	[Wang et al 2017]
**OTHERS**			
nicotinic acid (niacin; vitamin B_3)	F↓	solvent extraction GC-TOFMS	[Phua et al 2014]
pantothenic acid (vitamin B_5)	F↑	solvent extraction GC-MS	[Weir et al 2013]
	F↓	solvent extraction GC-MS	[Wang et al 2017]

where: ↑—concentration elevated in comparison of healthy controls; ↓—concentration decreased in comparison of healthy controls; post—index regarding postoperative samples; m—index regarding only male samples; no arrows—changes of concentration of compound not mentioned by authors; GC-MS—gas chromatography-mass spectrometry; dHS-SPME-GC-qMS—dynamic headspace solid-phase microextraction gas chromatography-quadrupole mass spectrometry; HS-SPME-GC-MS—headspace solid-phase microextraction gas chromatography-mass spectrometry; TD-GC-MS—thermal desorption gas chromatography-mass spectrometry; GC-FID—gas chromatography-flame ionization detector; GC-TOFMS—gas chromatography-time-of-flight mass spectrometry; U—urine; F—feces; Br—breath.
